# Probing Bethe strings in an attractive one-dimensional Bose gas

**DOI:** 10.1038/s41467-026-76018-0

**Published:** 2026-08-29

**Authors:** Milena Horvath, Alvise Bastianello, Sudipta Dhar, Rebekka Koch, Yanliang Guo, Jean-Sébastien Caux, Manuele Landini, Hanns-Christoph Nägerl

**Affiliations:** 1 Institut für Experimentalphysik und Zentrum für Quantenphysik, Universität Innsbruck, Technikerstraße 25, Innsbruck, Austria; 2 Technical University of Munich, TUM School of Natural Sciences, Physics Department, Garching, Germany; 3 Munich Center for Quantum Science and Technology (MCQST), Schellingstr. 4, München, Germany; 4 Institute of Physics and Institute for Theoretical Physics, University of Amsterdam, PO Box 94485, Amsterdam, The Netherlands

**Keywords:** Quantum simulation, Bose-Einstein condensates

## Abstract

Bethe strings are bound states of constituent particles in a variety of interacting many-body one-dimensional (1D) integrable quantum models relevant to magnetism, nanophysics, cold atoms and beyond. They are the quantum analogue to bright solitons. As emergent fundamental excitations, they are predicted to collectively reshape observable equilibrium and dynamical properties. Small individual Bethe strings have recently been observed in quantum magnets and superconducting qubits. However, creating states featuring intermixtures of many, including large, strings remains an outstanding experimental challenge. Here, using nearly integrable ultracold Bose gases, we realize such intermixtures of Bethe strings out of equilibrium, by dynamically tuning interactions from repulsive to attractive. We measure the average binding energy of the strings, revealing the presence of bound states of more than six particles. We find further evidence for them in the momentum distribution and in Tan’s contact, connected to the correlated density. Our data quantitatively agree with predictions from generalized hydrodynamics (GHD). Manipulating intermixtures of Bethe strings opens new avenues for understanding quantum coherence, nonlinear dynamics and thermalization in strongly-interacting 1D systems.

## Abstract (web summary)

Despite several studies reporting Bethe string states of one-dimensional quantum models realized in different platforms, their experimental observation with strongly interacting Bose gases is elusive. Here, the authors demonstrate Bethe string states in strongly interacting out of equilibrium Bose gases.

## Introduction

Strong correlations present modern physics with both challenges and opportunities. In elementary cases, interactions merely renormalize bare excitations^1^ or generate emergent collective hydrodynamic modes^2,3^. A further (and far more exciting) possibility is for a non-perturbative overhaul of microscopic degrees of freedom to occur, invalidating any description of the many-body state from a non-interacting basis. The formation of bound states is a simple manifestation of this paradigm: Bardeen-Cooper-Schrieffer (BCS) pairs illustrate how bound states give rise to superconductivity^4^, while quark confinement leads to the hadrons of high-energy physics^5^. Bosonic systems are potentially far richer than fermionic ones, since they accommodate large compounds of tightly-bound and thus strongly-correlated emergent particles. Realizing long-lived ensembles of attractive bosons, however, poses major practical challenges in analogy with the vacuum instability of attractive QED^6^. In bosonic systems, the absence of Pauli exclusion permits the formation of large complexes, which in turn collapse due to short-ranged molecular processes. Attractively interacting bosons have thus been observed only at weak interactions for a limited number of particles ^7–9^, or in one dimension for strong attractive interactions^10–14^ where bound states are suppressed due to energetics. The realization of a system of strongly attractive bosons displaying hierarchies of large bound states has remained elusive due to these difficulties.

In 1D integrable quantum systems^15^, attractive interactions do not necessarily induce collapse, but instead give rise to Bethe strings, multi-particle bound states predicted nearly a century ago^16^. They emerge as stable excitations in spin chains^15^, cold atomic gases^17^, and quantum magnets^18,19^. Their stability stems from the characteristic absence of diffractive collisions in integrable systems^15^, which prevents bound states from decaying into lower-lying states. Recent studies show that large Bethe strings determine anomalous transport properties of integrable spin chains and drive the emergence of the Kardar-Parisi-Zhang (KPZ) universality class^20–27^. Beyond traditional condensed matter systems, they are also relevant in high-energy physics and string theory^28^. A proper understanding of this wealth of manifestations requires the ability to create and manipulate dense intermixtures of Bethe strings of different sizes.

Bethe strings are expected to exist in the cold-atom Lieb-Liniger (LL) setting^29,30^. In the semiclassical limit, Bethe strings converge to the bright solitons of the nonlinear Schrödinger equation ^31^. Soliton-trains, stable only at weak interactions, have been realized in attractive bosons near the semiclassical regime^9^. Soliton formation is understood in this context as modulation instability of the non-linear Schrödinger equation and its generalizations^32^. Here, using ultracold attractively-interacting Cs atoms in 1D, we create a dense intermixture of strings by sweeping the interaction from repulsive to attractive, passing through the non-interacting point, as proposed in Ref. ^33^. We operate in the quantum regime beyond the applicability regime of the non-linear Schrödinger equation, and a fast collapse of the gas is hindered by the approximate integrability^29,30^ of the system. Using a finite-rate sweep, we create a far-from-equilibrium state that exhibits multiple, including large, Bethe strings. Their typical size is set by the initial conditions, primarily the temperature and atomic cloud density. We detect their signature by comparing release measurements in 1D and 3D and by analyzing the momentum distribution and performing a measurement of Tan’s contact^34^. Our data agrees well with the predictions^33,35^ from generalized hydrodynamics^36,37^.

## Results

**Fig. 1:**
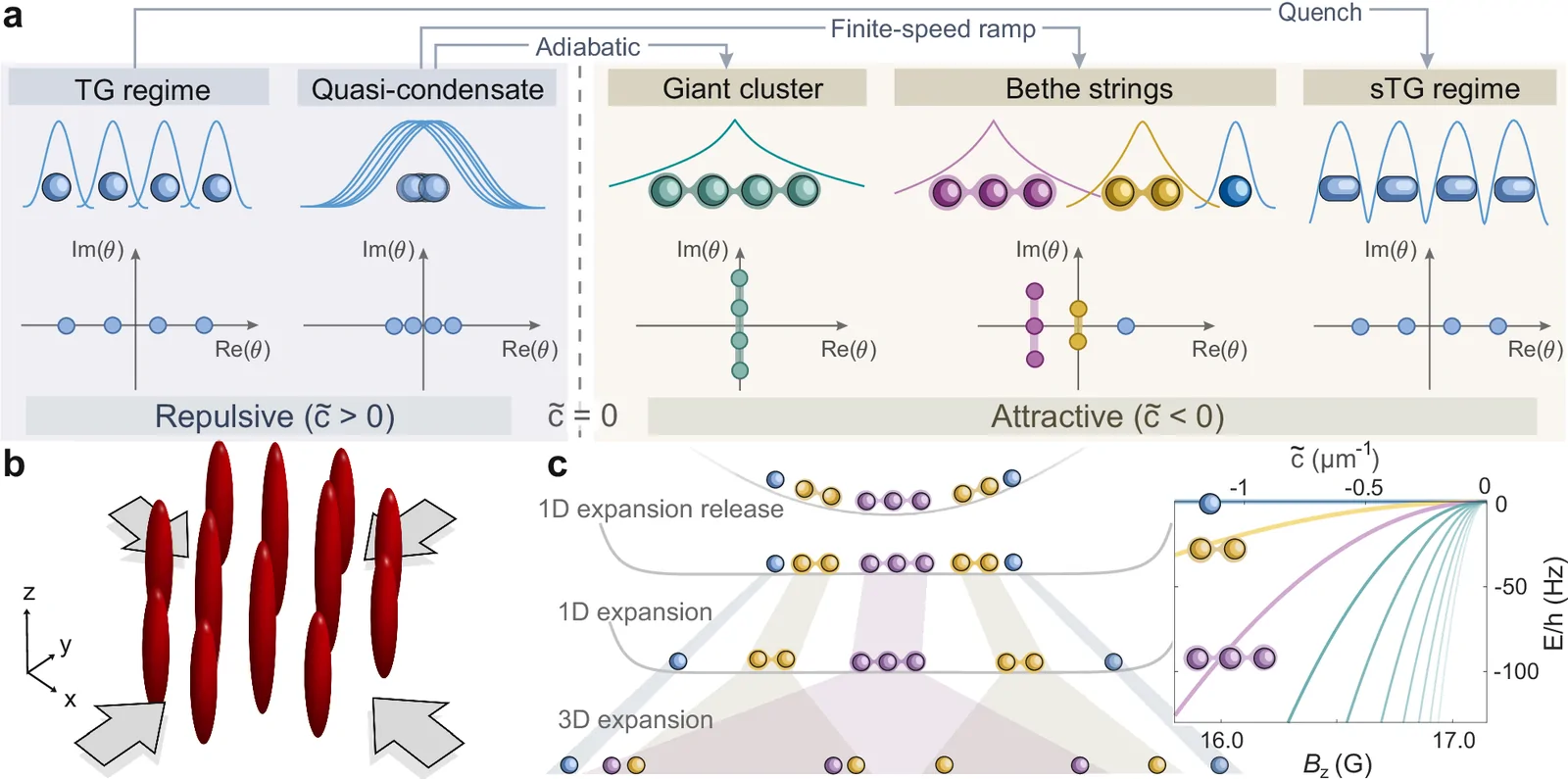
Bethe strings and their detection in 1D Bose gases. **a** Pictorial representation of the different interaction regimes of 1D Bose gases. Wavefunction correlation of the different states (top panel) and corresponding rapidity distribution *θ* (bottom panel). As the interaction parameter $\tilde{c}$ is increased from 0 (quasi-condensate) to +*∞* (TG regime), the gas fermionizes. The sTG regime can be accessed by quenching the interaction from the repulsive to the infinitely attractive regime. In contrast, adiabatically following the ground state of the system from positive to negative interactions $\tilde{c}$, and passing through $\tilde{c}=0$, results in a giant cluster. Sweeping $\tilde{c}$ from the strongly repulsive regime through the $\tilde{c}=0$ point with a finite rate realizes a non-equilibrium 1D gas composed of Bethe strings. Bound states with different numbers of particles are represented by different colors. **b** Illustration of the experimental geometry. Horizontally propagating lattice beams (gray arrows) create an ensemble of independent 1D Bose gases (red tubes). **c** Schematic of the 1D-3D expansion protocol used to probe Bethe strings. In the 1D, the expansion, Bethe strings remain bound, breaking apart only upon the 3D release. Inset: Energy of different string states as a function of both $\tilde{c}$ and *B*_*z*_, for bound states with up to ten particles.

Bethe strings arise as eigenstates of the celebrated LL Hamiltonian

1 $${\hat{H}}_{{{{\rm{LL}}}}}=-\frac{{\hslash }^{2}}{2m}{\sum}_{i}\frac{{\partial }^{2}}{\partial {z}_{i}^{2}}+\frac{{\hslash }^{2}\tilde{c}}{2m}{\sum}_{i\ne j}\delta ({z}_{i}-{z}_{j}),$$

where the first term is the kinetic energy for the particles with mass *m* and the second term models the inter-particle interactions with strength $\tilde{c}$. The eigenstates, obtained by Bethe ansatz^16,29^, are labeled by a set of rapidities *θ*^15^, which are the generalized momenta of a set of emergent, stable quasiparticles. Figure 1a shows a schematic representation of the different interaction regimes of the 1D Bose gas. For $\tilde{c} > 0$, the particles interact repulsively, and the rapidities are real quantities. For sufficiently strong repulsive interactions, the bosons fermionize in the Tonks-Girardeau (TG) state^38,39^. A quench to strong attractive interactions ($\tilde{c} < 0$) realizes an excited gaseous state, the super Tonks-Girardeau (sTG) state^10^. However, in the attractive regime, the rapidities are generally complex valued with non-zero imaginary parts. They are arranged in clusters that share the same real part *λ* ≡ Re(*θ*), symmetrically placed along the imaginary axis with spacing $| \tilde{c}|$. These states are the Bethe strings^40,41^. An isolated Bethe string of size *n* has the wavefunction $\psi ({z}_{1} < \ldots < {z}_{n})\propto \exp \left(i{\sum }_{j=1}^{n}{\theta }_{j}{z}_{j}\right)$, with rapidities ${\theta }_{j}=\lambda+i| \tilde{c}| (n+1-2j)/2$. Its extent Δ*z*_*n*_ can be estimated from the wavefunction decay as ${{\Delta }}{z}_{n}=2{[| \tilde{c}| (n-1)]}^{-1}$, where the length scale is set by $| \tilde{c}{| }^{-1}$. The total energy *E*_*n*_ of the string is *E*_com;n_ − *E*_b;n_, where *E*_com;n_ = *nℏ*^2^*λ*^2^/(2*m*) is the center-of-mass energy and ${E}_{{{{\rm{b;n}}}}}={\hslash }^{2}{\tilde{c}}^{2}n({n}^{2}-1)/(24m)$ is the binding energy^30^. The very ground state for *N* particles is a giant Bethe state with *N* constituents. Bethe strings with a lesser number of constituents have to be realized in a non-equilibrium situation^33,42,43^, here realized with a finite-rate interaction ramp from the repulsive to the attractive phase, passing through the non-interacting point. The specifics of the initial state, the ramp rate, and the necessarily nonzero temperature will then determine the distribution of Bethe states in such a non-equilibrium situation.

## Experimental protocol

Our experimental sequence starts with a 3D Bose-Einstein condensate (BEC) of Cs in a crossed-beam optical dipole trap, levitated against gravity by a magnetic force^44^. We control the atom number in the BEC within the range of 2 × 10^4^ to 6 × 10^4^, with an uncertainty of 10%, and the temperature between 10 nK to 35 nK, with a precision of 1 nK. A broad Feshbach resonance allows us to tune the 3D s-wave scattering length *a*_3D_ via an offset magnetic field *B*_*z*_. Initially, *B*_*z*_ is set to 20.8(1) G, at which *a*_3D_ = 209(4) *a*_0_. The BEC is in the Thomas-Fermi regime. It is adiabatically loaded into two lattice beams that cross at 90^∘^ in the horizontal *x* − *y* plane (see Fig. 1b). This results in an array of isolated 1D tubes with trapping frequencies of *ω*_⊥_/2*π *= 10.5(1) kHz in the transversal and *ω*_*z*_/2*π* = 29.3(1) Hz in the longitudinal direction for a lattice depth of 25 *E*_r_, where, *E*_r_ = *π*^2^*ℏ*^2^/(2*md*^2^) is the recoil energy and *d *= 532.2 nm is the lattice spacing set by the wavelength of the lattice light. This gives longitudinal and transversal harmonic oscillator lengths of *a*_ho_ = 1.6(2) μm and *a*_⊥_ = 83(4) nm, respectively. We tune the average number of atoms per tube *N* between 7 and 30 by varying the density of the initial BEC, resulting in 3000 to 5000 populated tubes.

In our 1D setting, the strength of the interaction is controlled via $\tilde{c}\simeq 4{a}_{{{{\rm{3D}}}}}/{a}_{\perp }^{2}$^45^. After lattice loading, we set the interaction strength to $\tilde{c}=3.4(1)$ μm^−1^, putting our systems in the moderately interacting TG regime. The 1D temperature *T*_1D_ is set to values between 3 nK and 35 nK with an uncertainty of 20% (see “Methods”). In this range of parameters, our system is in the quantum regime^46^. There, the system cannot be described within the Gross-Pitaevskii approximation^47^, and GHD appears as the only viable theoretical approach. We ramp the interaction parameter to lower and to negative values, crossing the non-interacting point, with a nearly constant rate of $\partial \tilde{c}/\partial t\approx -0.038$ μm^−1^ms^−1^. We expect that this procedure maps our system onto a distribution of Bethe strings as the interactions become attractive^33^. For our experiment, for an interaction strength of $\tilde{c}=-1$ μm^−1^, a string of size *n *= 4 has a bond length of about 0.67 μm. The binding energy of this string then is *h* × 190 H ≈ *k*_B_ × 9 nK. Note that Bethe strings are only well defined when their bond length Δ*z*_*n*_ is much larger than *a*_⊥_. For short distances, loss through short-range molecular processes is expected. Such a loss is not captured by the LL model. The absence of transverse mode excitations in time-of-flight (TOF) measurements confirms that the system remains deeply in the 1D regime (see “Methods”).

We employ the rapidity measurement protocol^13,48–51^, previously used in the absence of bound states, to probe our system. This protocol consists of a two-step expansion sequence: first, an expansion within the tubes for a time *t*_1D_, followed by a free expansion into 3D space for time *t*_3D_ (see Fig. 1c). We ensure that *t*_1D_ is large enough so that the system enters a dilute regime, where the interparticle interactions become negligible (see “Methods”). In the repulsive regime, a subsequent absorption image yields the rapidity distribution of the gas. However, in the attractive regime, the connection between the rapidity distribution and the absorption image becomes more intricate, as we explain below. Experimentally, the expansion in the tubes is achieved by simultaneously switching off the dipole traps and removing the residual longitudinal confinement created by the lattice beams by applying a 808-nm blue-detuned anti-trapping beam that flattens out the potential^52^. For the second expansion step, the expansion into 3D, we simply shut off the transverse confinement and simultaneously set *a*_3D_ ≃ 0 to avoid any interaction-induced broadening of the density distribution of the expanding cloud.

## Release energy measurements

**Fig. 2:**
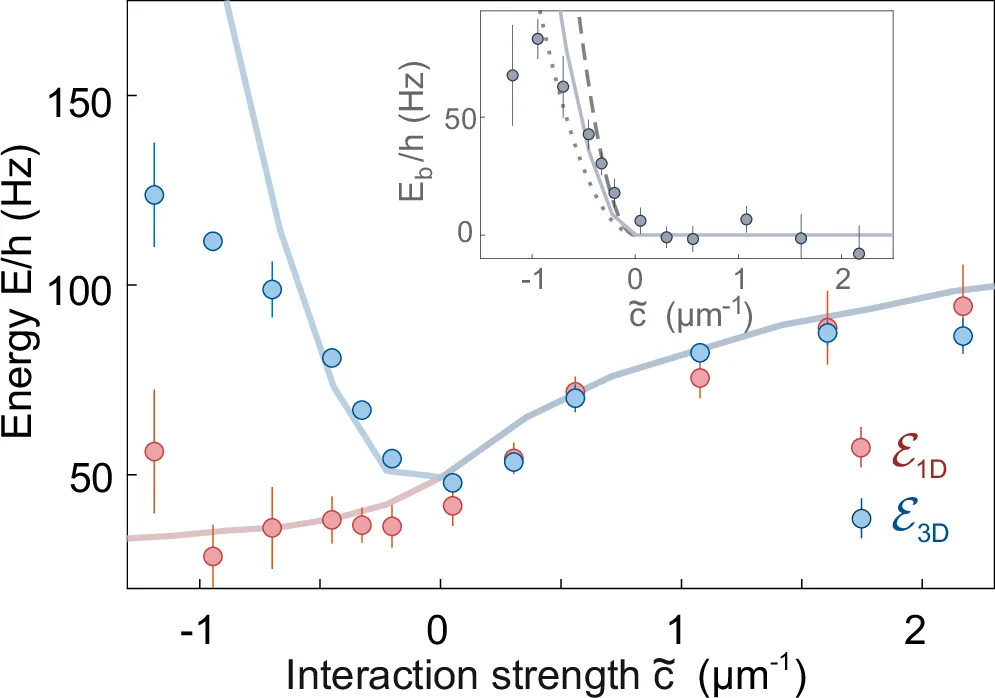
Evidence for Bethe strings from release measurements. Release energy per particle ${{{{\mathcal{E}}}}}_{{{{\rm{1D}}}}}$ (red) and ${{{{\mathcal{E}}}}}_{{{{\rm{3D}}}}}$ (blue) as a function of the interaction parameter $\tilde{c}$ at the end of the ramp. Each data point for the ${{{{\mathcal{E}}}}}_{{{{\rm{1D}}}}}$ (${{{{\mathcal{E}}}}}_{{{{\rm{3D}}}}}$) energy is determined from a fit of the change of the second moment of the cloud as a function of *t*_1D_ (*t*_3D_). The initial 1D temperature is estimated to be *T*_1D_ = 7 nK (see “Methods”), and *N* = 11. Inset: The average binding energy obtained via $\langle {E}_{{{{\rm{b}}}}}\rangle={{{{\mathcal{E}}}}}_{{{{\rm{3D}}}}}-{{{{\mathcal{E}}}}}_{{{{\rm{1D}}}}}$. The dotted and dashed curves correspond to the binding energy per particle of the Bethe strings of sizes *n* = 6 and 10, respectively. For both plots, the standard error is given. The solid curves correspond to results from GHD, accounting for the full probability distribution of string sizes^33^.

In the first experiment, we aim to find evidence of Bethe strings via measurements of the release energy. We extract the release energies ${{{{\mathcal{E}}}}}_{{{{\rm{1D}}}}}$ and ${{{{\mathcal{E}}}}}_{{{{\rm{3D}}}}}$ from the evolution of the second moment of the expanding cloud during 1D and 3D phases of our rapidity measurement protocol. ${{{{\mathcal{E}}}}}_{{{{\rm{1D}}}}}$ is obtained by varying *t*_1D_ while keeping *t*_3D_ constant, and ${{{{\mathcal{E}}}}}_{{{{\rm{3D}}}}}$ is obtained by varying *t*_3D_ while keeping *t*_1D_ constant (see “Methods”). Figure 2 shows ${{{{\mathcal{E}}}}}_{{{{\rm{1D}}}}}$ and ${{{{\mathcal{E}}}}}_{{{{\rm{3D}}}}}$ as a function of $\tilde{c}$. We find that for the repulsive case, ${{{{\mathcal{E}}}}}_{{{{\rm{1D}}}}}$ and ${{{{\mathcal{E}}}}}_{{{{\rm{3D}}}}}$ match within the error, and decrease from about *h* × 100 Hz in the strongly repulsive side to approximately *h* × 60 Hz close to the non-interacting point. In contrast, a clear difference can be detected for attractive interactions. While ${{{{\mathcal{E}}}}}_{{{{\rm{1D}}}}}$ plateaus around *h* × 35 Hz, ${{{{\mathcal{E}}}}}_{{{{\rm{3D}}}}}$ increases to about *h* × 110 Hz for $\tilde{c}=-1.0$ μm^−1^. This discrepancy between ${{{{\mathcal{E}}}}}_{{{{\rm{1D}}}}}$ and ${{{{\mathcal{E}}}}}_{{{{\rm{3D}}}}}$ for attractive interactions provides strong evidence for the presence of Bethe strings. During the 1D expansion, these bound states scatter, but remain intact, leading to a measured ${{{{\mathcal{E}}}}}_{{{{\rm{1D}}}}}$ that remains low. However, upon release into 3D, the sudden removal of the confinement breaks the bound states, converting their binding into kinetic energy, which leads to a larger release energy, mainly in the *z*-direction. In the repulsive regime, on the other hand, the absence of bound states ensures that ${{{{\mathcal{E}}}}}_{{{{\rm{1D}}}}}$ and ${{{{\mathcal{E}}}}}_{{{{\rm{3D}}}}}$ are equal. In fact, in the repulsive regime, ${{{{\mathcal{E}}}}}_{{{{\rm{1D}}}}}={{{{\mathcal{E}}}}}_{{{{\rm{3D}}}}}=U$, where $U=\langle {\hat{H}}_{{{{\rm{LL}}}}}\rangle$ is the internal energy of the gas. In the attractive regime, ${{{{\mathcal{E}}}}}_{{{{\rm{1D}}}}}$ is given by the average center-of-mass energy of the strings ${{{{\mathcal{E}}}}}_{{{{\rm{1D}}}}}=\langle {E}_{{{{\rm{com}}}}}\rangle$. In contrast, ${{{{\mathcal{E}}}}}_{{{{\rm{3D}}}}}$ is the contribution from both the average center-of-mass energy of the strings and the average binding energy ${{{{\mathcal{E}}}}}_{{{{\rm{3D}}}}}=\langle {E}_{{{{\rm{com}}}}}\rangle+\langle {E}_{{{{\rm{b}}}}}\rangle$ (see Supplementary Information). The corresponding GHD simulations agree well with our measurements. Despite the finite number of particles, GHD evidently provides a quantitative description of the experiment, as previously noticed in the repulsive regime^26^. The GHD simulations account for the inhomogeneous trapping potential and for finite TOF effects by simulating the entire 1D and 3D expansions performed in the experiment (see Supplementary Information). The dominant integrability-breaking process in our experiment is three-body loss, which is not captured by GHD. Such loss is negligible in the repulsive phase, but in the attractive regime, as Bethe strings form and tighten upon increasing interactions, three-body losses become appreciable (see “Methods”). Large Bethe strings are lost for stronger attractive interactions, as they are more tightly bound. This picture qualitatively explains the discrepancy between experimental data and GHD in Fig. 2.

In the inset of Fig. 2 we plot 〈*E*_b_〉 as a function of $\tilde{c}$ obtained via ${{{{\mathcal{E}}}}}_{{{{\rm{3D}}}}}-{{{{\mathcal{E}}}}}_{{{{\rm{1D}}}}}$. Without resorting to advanced theoretical modeling within GHD, we can extract from the experimental data a lower bound for the maximum Bethe string size present in the system. The Bethe strings’ binding energy monotonically increases with the number of its components. To account for the observed value of 〈*E*_b_〉, the system necessarily contains strings of size n^*^ or larger, where n^*^ is the largest integer such that $\langle {E}_{{{{\rm{b}}}}}\rangle \ge {E}_{{\mbox{b}},{n}^{*}}/{n}^{*}$. This rough estimate results in n^*^ of at least 6, but a more refined analysis through GHD (see Supplementary Information) predicts larger Bethe strings to be also excited, as we discuss below.

## Characterization of 1D expansion

**Fig. 3:**
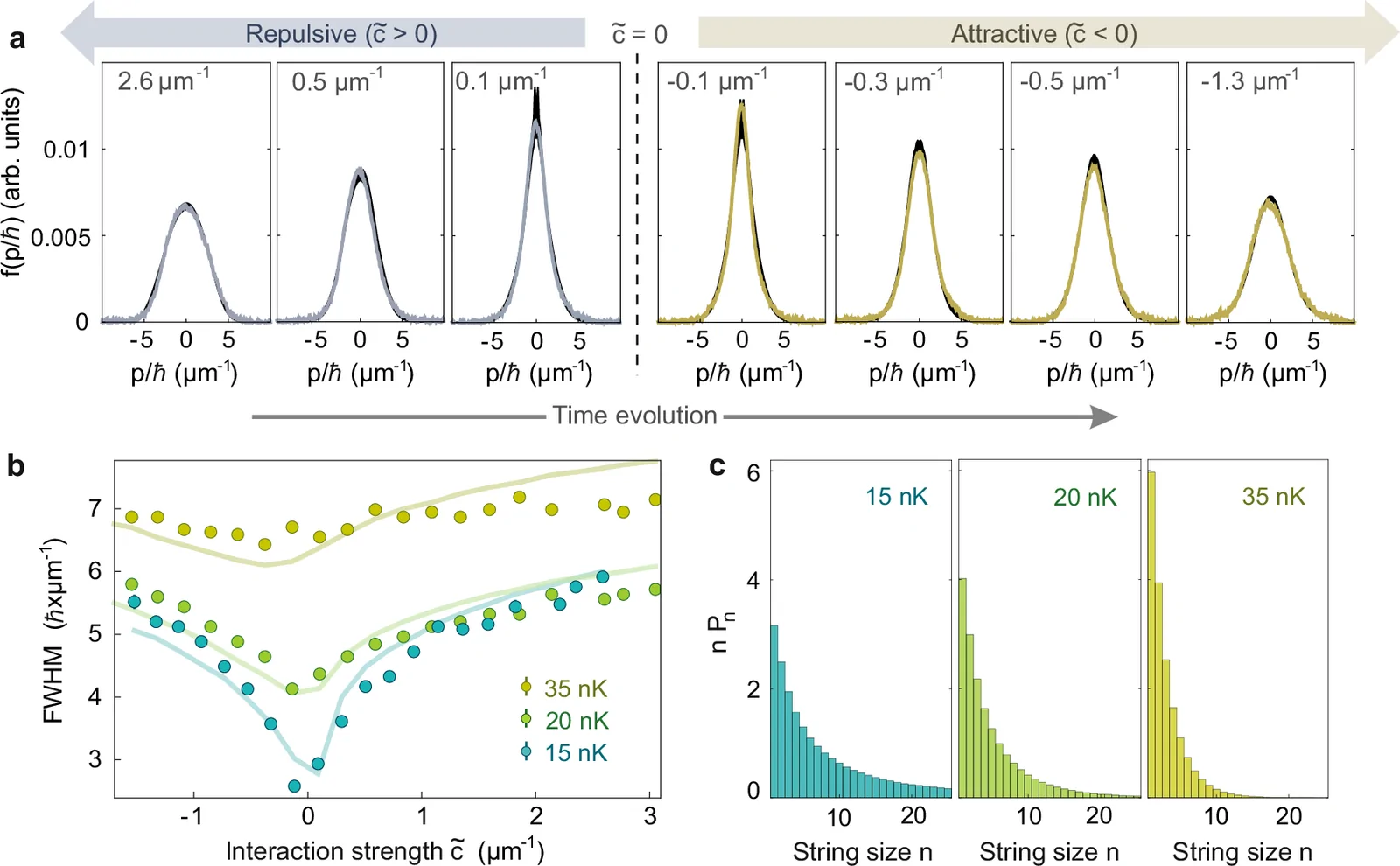
Evidence for Bethe strings from the momentum distribution via a comparison with the results of GHD. **a** Momentum distribution after 1D expansion *f*(*p*/*ℏ*) for various values of the interaction parameter $\tilde{c}$ at the end of the ramp as indicated, from the repulsive (left, blue) to the attractive (right, yellow) regime. Each distribution is the mean of five repetitions, and the shaded region corresponds to the standard error. The black shaded region corresponds to the results from GHD assuming an initial 1D temperature of *T*_1D_ = 15 nK with an error of ±10%. **b** FWHM of *f*(*p*/*ℏ*) as a function of $\tilde{c}$ for three selected values of the *T*_1D_ as indicated in the figure. Each data point is the average of five repetitions. The standard error is given. **c** Tube-averaged average number of atoms *nP*_*n*_ that participate in strings of size *n* as predicted by theory for the temperatures as in Fig. 3b.

We find further evidence for Bethe strings by comparing the momentum distribution after 1D expansion *f*(*p*/*ℏ*) with the predictions of GHD. For this, we fix *t*_1D_ = 10 ms and *t*_3D_ = 46.3 ms. The momentum distribution is obtained from the longitudinal profile of the expanded cloud, converting position *z* to momentum as *p* = *mz*/(*t*_1D_ + *t*_3D_). In Fig. 3a, we show *f*(*p*/*ℏ*) as the interaction strength $\tilde{c}$ is ramped from the repulsive to the attractive regime. At the beginning of the ramp, *f*(*p*/*ℏ*) is bell-shaped with a full width at half maximum (FWHM) *ℏ* × 6.0(1) μm^−1^. As $\tilde{c}$ decreases and approaches the non-interacting point, *f*(*p*/*ℏ*) becomes more sharply peaked, with the FWHM narrowing to *ℏ* × 3.0(1) μm^−1^ at $\widetilde{c}=0.1$ μm^−1^. After crossing the zero-interaction point, *f*(*p*/*ℏ*) broadens, nearly doubling in size by the end of the ramp at $\tilde{c}=-2.0$ μm^−1^. Next, we compare our data with GHD. We estimate the best initial conditions for the simulations by optimizing the match with experimental data in the weakly repulsive regime, which is most sensitive to the initial parameters (see “Methods”). This results in *T*_1D_ = 15 nK. We find good agreement between the experimental data and the results from GHD across the whole interaction regime. Only minor corrections close to the non-interacting point are observed when varying the initial temperature in the GHD simulation by ±10%, reflecting the robustness of the GHD results. In the repulsive regime, *f*(*p*/*ℏ*) corresponds to the rapidity distribution of the gas (see Supplementary Information). Therefore, the narrowing of the distributions for decreasing $\tilde{c} > 0$ is attributed to a reduction in the effective velocity of the quasiparticles. In contrast, for the attractive case $\tilde{c} < 0$, the quasiparticles bind together to form Bethe strings that broaden the distributions. An intuitive understanding based on Heisenberg’s uncertainty principle suggests that a string with bond length Δ*z*_*n*_ will release momentum within a window Δ*p*_*n*_ ~ /(2Δ*z*_*n*_). This broadening effect becomes more prominent for larger Bethe strings and for stronger attractive interactions.

We now examine how the temperature affects the formation of the bound states. In Fig. 3b, we show the FWHM of *f*(*p*/*ℏ*) as a function of $\tilde{c}$ for three different values of *T*_1D_. Note that for the 15 nK dataset, we use a lattice depth of 20 E_r_ instead of 25 E_r_. As is already evident from Fig. 3a the distribution broadens away from $\tilde{c}=0$. This effect diminishes for higher *T*_1D_. Results from GHD are in good agreement with our experimental data. We attribute the larger discrepancy of the higher-temperature data to the less accurate description of the initial conditions with our two-parameter model (see “Methods”). Figure 3c shows the GHD prediction for the number of atoms *nP*_*n*_ that are bound in strings of size *n*, where *P*_*n*_ is the probability of finding such a string. The probability of forming larger strings decreases with increasing *T*_1D_. For increasing temperature, the kinetic energy of the system dominates over the interaction energy. This reduces the probability of forming larger bound states and, in turn, reduces the average binding energy per particle. This is reflected in Fig. 3b, where the FWHM of *f*(*p*/*ℏ*) becomes less sensitive to the change of interaction at higher *T*_1D_.

## Tan’s contact

The presence of Bethe strings significantly modifies the short-range correlations of the gas, notably the local pair correlations integrated over the trap ${G}_{2}=\langle {\sum}_{i\ne j}\delta ({z}_{i}-{z}_{j})\rangle$^53^, which quantify the probability of finding two particles in the same place. This is directly proportional to Tan’s contact $C={\tilde{c}}^{2}{G}_{2}$^34,54^. Tan’s contact establishes a fundamental link between microscopic quantities and the thermodynamic properties of the system. At thermal equilibrium, the virial theorem ^55^ relates *C* to the internal energy *U* and the potential energy *E*_V_ of the harmonic trap as

2 $$C=\frac{2m\tilde{c}}{{\hslash }^{2}}(U-{E}_{{{{\rm{V}}}}}).$$

We find that Eq. (2) also generalizes to out-of-equilibrium scenarios described by stationary solutions for GHD (see Supplementary Information).

**Fig. 4:**
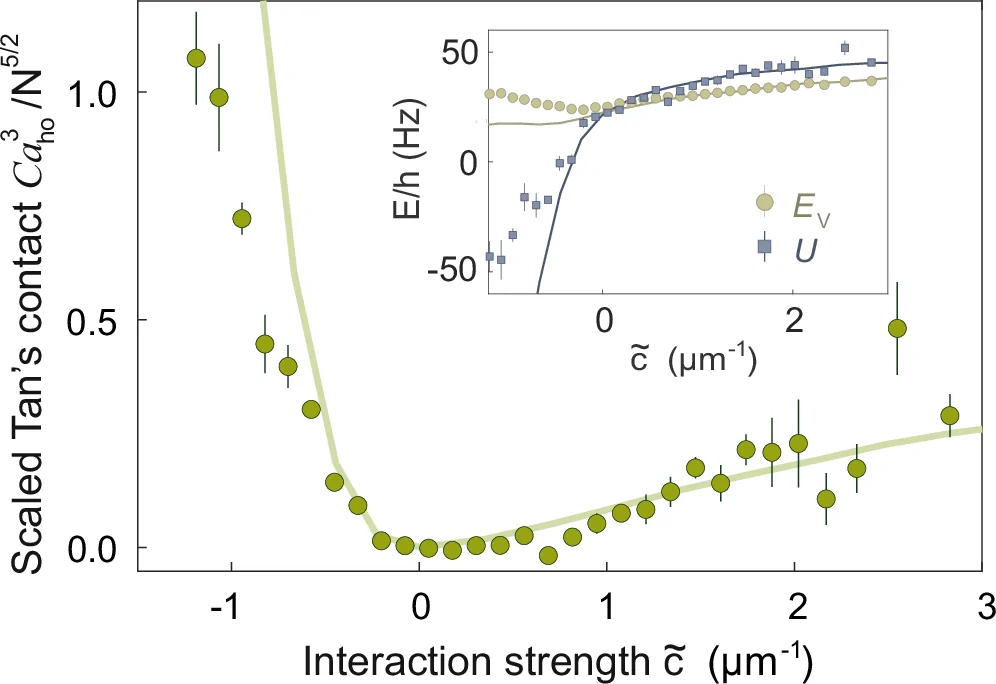
Interaction dependence of Tan’s contact. The scaled contact (circles) is extracted from experimental measurements of *U* and *E*_*V*_ via Eq. (2). Inset: The potential energy *E*_V_ (circles) and internal energy *U* (squares) as a function of $\tilde{c}$. The solid curves in both plots are results obtained from GHD. For these measurements *T*_1D_ = 3 nK and *N* = 5. The standard error is given.

Experimentally, we obtain *U* from the 3D release-energy measurements described previously. While for repulsive interactions we can directly extract *U* from ${{{{\mathcal{E}}}}}_{{{{\rm{3D}}}}}$, for attractive regime this is not the case. As shown in Fig. 2, 〈*E*_com_〉 plateaus within experimental uncertainty for attractive interactions. We therefore approximate $\langle {E}_{{{{\rm{com}}}}}\rangle (\tilde{c} < 0)$ $\simeq {{{{\mathcal{E}}}}}_{{{{\rm{3D}}}}}(\tilde{c}=0)$. The binding energy 〈*E*_b_〉 is given by ${{{{\mathcal{E}}}}}_{{{{\rm{3D}}}}}-{{{{\mathcal{E}}}}}_{{{{\rm{3D}}}}}(\tilde{c}=0)$. We use this approximation to extract *U* for the attractive regime. The potential energy is measured by imaging the spatial distribution of the atom cloud. We use a short TOF of 1.5 ms without 1D expansion in order to lower the optical density before imaging. The potential energy per particle is given by ${E}_{{{{\rm{V}}}}}=(1/2)m{\omega }_{z}^{2}\langle {z}^{2}\rangle$, where 〈*z*^2^〉 is the mean squared width of the cloud in the longitudinal direction. In Fig. 4, we plot the interaction dependence of the contact, determined from the measured *U* and *E*_V_ using Eq. (2) (see inset of Fig. 4). On the repulsive side, Tan’s contact decreases as $\tilde{c}$ is reduced. Upon entering the attractive regime, Tan’s contact increases dramatically. It is nearly six times higher on the attractive side at the same magnitude of the interaction $| \tilde{c}|=1$ μm^−1^. We attribute this to the presence of Bethe strings as they increase the correlated density *G*_2_, their effect being more prominent as they become tighter with increasing interactions. The GHD predictions qualitatively describe experimental observations with very good agreement on the repulsive side. For $\tilde{c} < 0$, we attribute the discrepancy to finite-size effects, as theory predicts a substantial contribution from Bethe strings larger than the average number of particles in the tubes. Additional discrepancies stem from atom loss in the experiment, with approximately 20% of the atoms being lost during the ramp for the strongest attractive interaction (see “Methods”). Our data can be used to benchmark other theoretical methods for calculating Tan’s contact in 1D^56–58^.

## Discussion

In summary, we have used 1D attractive Bose gases to realize novel non-equilibrium states of quantum matter featuring dense intermixtures of Bethe strings. In our experiment, approximate integrability stabilizes these bound states and bypasses the rapid collapse normally associated with attractive bosons^7–9^. We have experimentally characterized these bound states through measurements of the binding energy, Tan’s contact, and the momentum distribution following 1D expansion. Our results agree with predictions from GHD, showcasing the latter’s validity beyond the previously-considered repulsive^46,49,50,59–61^ and sTG regimes^13^. Our results show that the presence of Bethe strings (somewhat similar to Efimov physics^62^) in correlated gases greatly enriches the landscape of achievable non-equilibrium states in the vicinity of integrability. Since Bethe strings display remarkable resilience to integrability breaking^63^, they can alter transport properties^64^, give rise to new interesting pre-thermal phases^65^, and affect the emergent hydrodynamics and thermalization of nearly-integrable systems^60,66–68^. Going further, it would be interesting to study their participation in the emergence of KPZ universality^69^. The fate of these Bethe strings in the dimensional crossover^70^ and the connection to and the onset of Efimov physics^71^ are also interesting avenues for future research.

## Methods

### Atom number estimation in tubes

Since the formation of Bethe strings in our protocol is a non-linear function of the density, having a good quantitative estimation of the distribution of atoms across the tubes is of key importance. Before the creation of the 1D tubes, we assume the gas is at thermal equilibrium, and it remains so until the dimensional crossover. When the transverse trapping is strong enough, the tubes cannot exchange particles any longer and are effectively decoupled. Just before the tubes decouple, we approximate the gas as a collection of 1D systems at thermal equilibrium, characterized by a unique temperature and a local chemical potential according to the shallow three-dimensional trap^72^. We also approximate the 1D interactions $\tilde{c}$ to be the same as the one at the end of the cross-over in one dimension. Below, we summarize in a table the relevant parameters for the tubes’ loading.

**Fig. 5:**
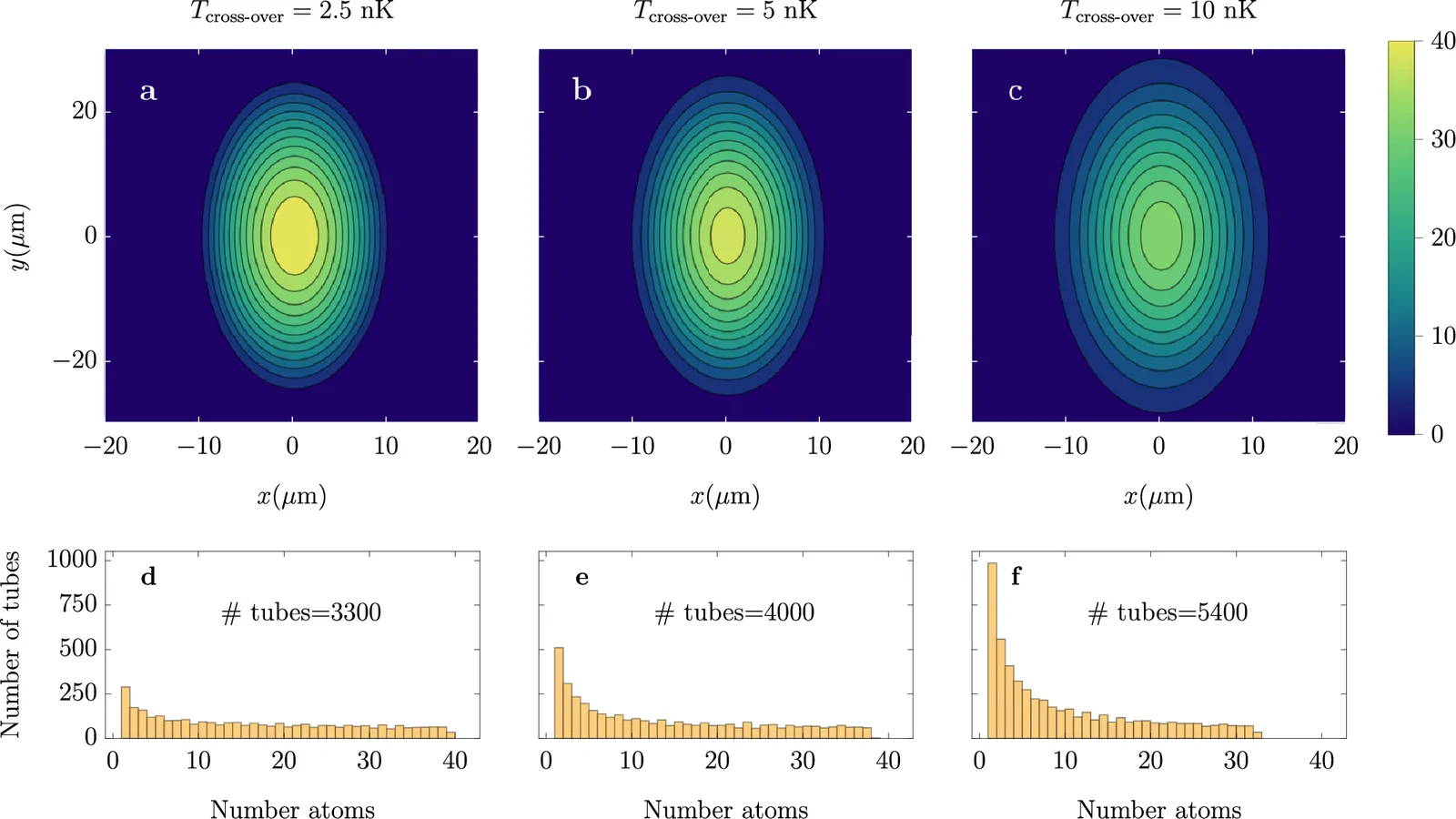
Example of tubes’ population varying *T*_cross-over_. As a concrete example, we show how tuning the dimensional crossover, the temperature changes the population of the tubes. In this example, we focus on the dataset of Fig. 3 corresponding to *T*_1D_ = 15nK. From left to right, the three columns correspond to the choices *T*_cross-over_ = (2.5, 5, 10) nK. The choice that best fits the initial data is *T*_cross-over_ = 5 nK. In the first row (**a**–**c**), we show the spatial distribution of the tube population in the (*x*, *y*) plane, whereas the longitudinal tubes are oriented in the *z* direction. By increasing *T*_cross-over_, more tubes are populated, but the average number of atoms in each of them decreases. This affects the creation of bound states when crossing to the attractive regime, as tubes with larger atom density populate larger bound states. In the second row (**d**–**f**), we show a histogram of the atom number population. Tubes with less than one atom are not accounted for, and “# tubes" is the total number of populated tubes in each case.

Under these assumptions, we can obtain the distribution *N*_*x*,*y*_ of the atomic sample across the array of tubes at position (*x*, *y*) using thermodynamic Bethe ansatz (see Supplementary Information), while keeping *T*_cross-over_ as a tunable parameter. Notice that during the dimensional crossover, the trapping frequency along the *z*− direction smoothly changes from the loading frequency *w*_1_ to the final 1D value. The thermodynamics of the 1D tubes at the dimensional crossover is computed using the loading frequency as the frequency of the 1D trap. An example of how different choices of *T*_cross-over_ affect the tubes’ population *N*_*x*,*y*_ is provided in Fig. 5. In this figure, the number of tubes corresponds to the tubes where *N*_*x*,*y*_ > 1. A working code solving the thermodynamics of the 1D tubes is provided on Zenodo^73^. From the dimensional crossover to the true 1D regime, the transverse trap is further increased, but the number of atoms in each tube remains constant. As the system enters deeper into the 1D regime, it approaches the integrable limit, hindering thermalization. We assume that at the end of the lattice loading protocol, each tube is still well-approximated by a thermal ensemble with a new temperature *T*_1D_, which is uniform across all the tubes. This approximation is currently the best modeling we can provide, but one could expect fluctuations in the 1D temperature across the tubes. Even more fundamentally, the approximation of considering a thermal Gibbs ensemble for the initial 1D tubes may not be exact, since in 1D, thermalization is suppressed. However, a quantitative modeling of these effects is beyond our current theoretical understanding; we assume the initial tubes are at thermal equilibrium with a uniform temperature *T*_1D_. The validity of this assumption is a posteriori supported by the good agreement with experimental data, which is particularly accurate at low temperatures with increasing corrections on hotter ensembles, see also Fig. 5. The parameters *T*_1D_ and *T*_cross-over_ are estimated by matching the experimental data in the weakly repulsive regime (see Supplementary Information). In Table 1, we summarize the experimental parameters and the corresponding results from the simulations for each of the datasets presented in the main text. The average atom number per tube *N* is obtained via

3 $$N=\frac{{\sum}_{x,y}{N}_{x,y}^{2}}{{\sum}_{x,y}{N}_{x,y}},$$

where *N*_*x*,*y*_ is the number of atoms in the tube at position (*x*, *y*).

**Table 1:** Experimental and theoretical parameters for each dataset are presented in the main text

	Experiment				Simulation			
Dataset	*N*_BEC_ (10^4^)	(*ω*_1_, *ω*_2_, *ω*_3_)/2*π* (Hz)	*ω*_*z*_/2*π* (Hz)	*ω*_⊥_/2*π* (kHz)	*T*_cross-over_ (nK)	*T*_1D_ (nK)	*N*	*N*_cent_
Fig. 2	3.2	(25.1, 10.1, 27.1)	29.3	10.5	10	7	11	21
Fig. 3	5.7	(25.1, 10.1, 27.0)	28.8	9.0	5	15	25	41
	5.7	(25.1, 10.1, 27.0)	27.6	10.5	10	20	19	34
	5.7	(25.1, 10.1, 27.0)	27.1	10.5	10	35	19	34
Fig. 4	1.9	(10.3, 5.7, 11.7)	18.1	11.5	3	3	5	9

For the experimental parameters, we give the initial atom number in the BEC *N*_BEC_, the lattice loading frequencies (*ω*_1_, *ω*_2_, *ω*_3_)/2*π* in the *z*, *x*, *y* directions, respectively. After lattice loading, the longitudinal *ω*_*z*_/2*π* and transversal *ω*_⊥_/2*π* trapping frequencies in the tubes are also indicated. From the simulation, we estimate the cross-over temperature *T*_cross-over_, the 1D temperature *T*_1D_, the average atom number per tube *N* and the central tube atom number *N*_cent_.

### Determining the release energies via the second moment

**Fig. 6:**
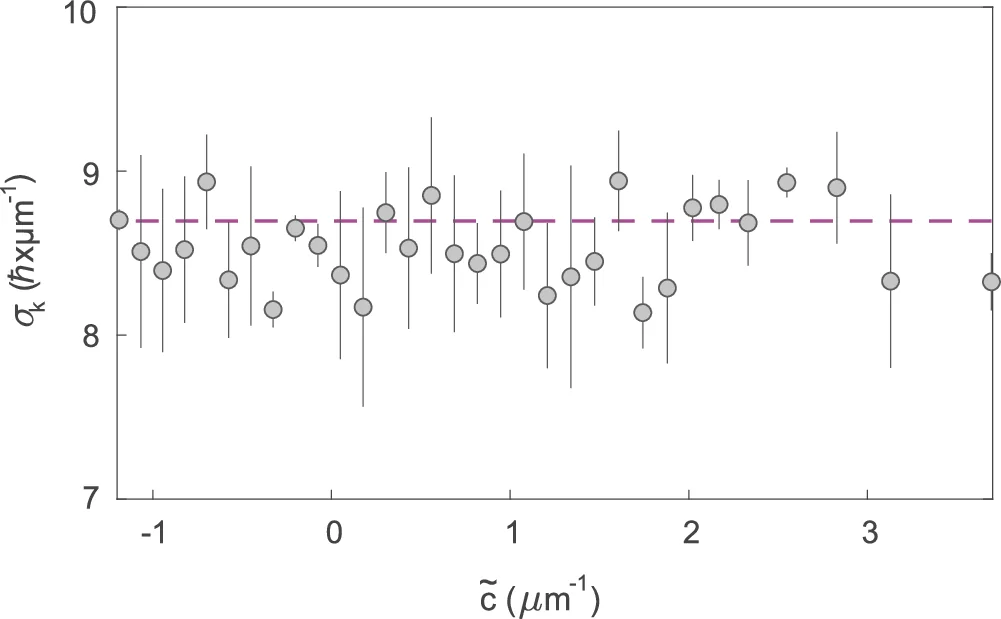
Absence of transverse excitations. Example transverse Gaussian width in momentum space, extracted from the dataset in Fig. 4, as a function of interaction strength for *t*_1D_ = 8, and *t*_3D_ = 22.1 ms. The dashed line indicates the expected width in momentum space assuming that the system is in the ground state.

**Fig. 7:**
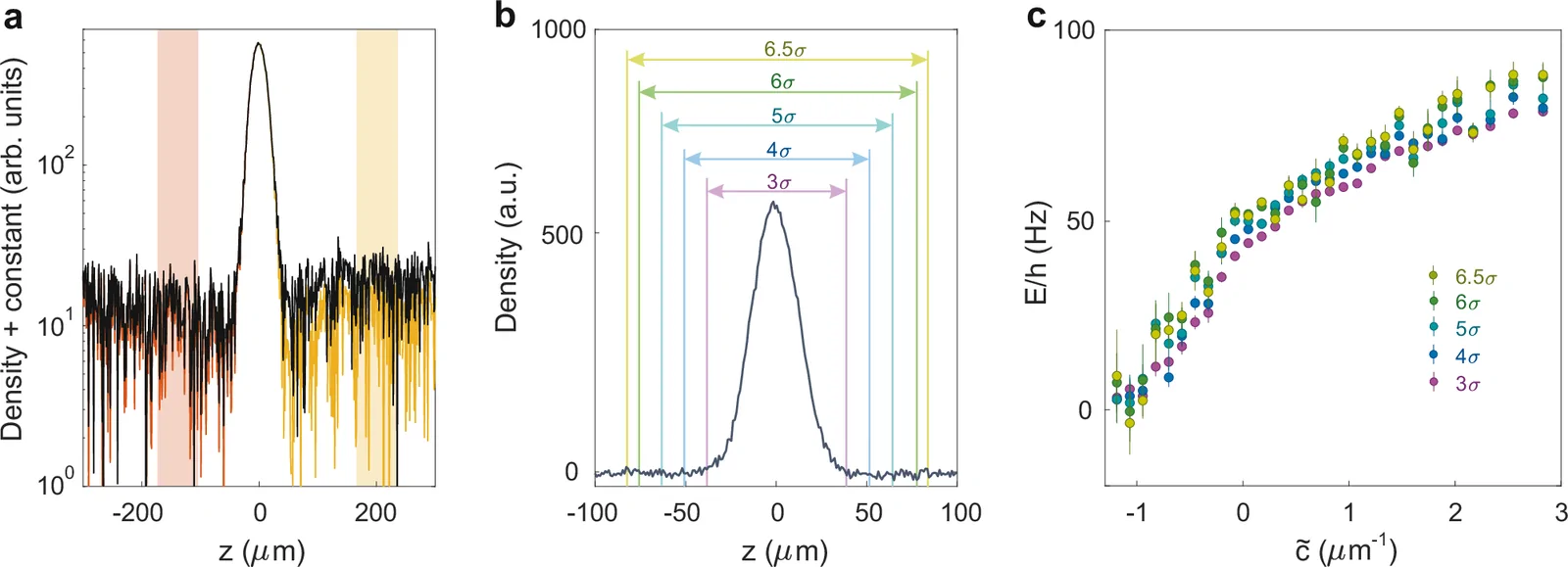
Convergence of energy extracted from the second moment of the distribution. **a** A typical TOF profile of the cloud in log-linear scale. *z* denotes the spatial position after TOF. For this plot, a small constant is added to the TOF distribution in order to avoid negative values in the logarithm. Different offsets are used on either side of the distribution in order to ensure that the second momentum converges for increasing regions of interest. Original data is shown in black, and the colored curves correspond to the data after the offset (mean over shaded region) has been applied. **b** Typical TOF distribution indicating different regions of interest used to obtain the internal energy *U* shown in (**c**). **c** Internal energy determined using the different regions of interest shown in (**b**).

We check that we remain in 1D throughout the interaction ramp protocol by fitting the transverse direction of the momentum distribution with a Gaussian function. Within experimental error, we observe a constant transverse width, indicating no transverse modes are populated throughout our protocol. A representative dataset for *t*_1D_ = 8 ms and *t*_3D_ = 24.5 ms is shown in Fig. 6. We determine the release energies ${{{{\mathcal{E}}}}}_{{{{\rm{1D}}}}}$ and ${{{{\mathcal{E}}}}}_{{{{\rm{3D}}}}}$ by measuring the expansion rate of the second moment of the sample 〈*z*^2^〉 during the 1D and 3D expansion stages, respectively. For the measurement shown in Fig. 2, we scan *t*_1D_ (*t*_3D_) from 6–11 ms (18–43 ms), while keeping *t*_3D_ = 20.4 ms (*t*_1D_ = 8 ms) fixed. For each experimental absorption image, we apply a background noise removal technique^74^ to improve the accuracy in the determination of the second moment. In Fig. 7a, we show a typical TOF density profile after noise removal. We find that far away from the signal, the distribution has a non-zero offset. Furthermore, this offset is different on either side of the distribution, which we attribute to the inhomogeneous profile of the imaging beam. Although the difference between the two offset values is less than 1%, it is crucial for determining the second moment 〈*z*^2^〉 of the distribution. In order to faithfully calculate the energy from 〈*z*^2^〉, we use different regions of interest (see Fig. 7b) and check for convergence in the estimated energy. As shown in Fig. 7c, we see a convergence in energy above the 3*σ* region of interest, where *σ* is the Gaussian width of the distribution. The energy is determined by fitting the evolution of 〈*z*^2^〉 as a function of expansion time. For the 1D release energy, we fit a parabolic function $a{({t}_{{{{\rm{1D}}}}}+{t}_{{{{\rm{fix}}}}})}^{2}+b$ to our 1D-expansion data, where *a* and *b* are fitting parameters, and *t*_fix_ is the fixed 3D expansion time. The fitting parameter *a* is related to ${{{{\mathcal{E}}}}}_{{{{\rm{1D}}}}}$ via ${{{{\mathcal{E}}}}}_{{{{\rm{1D}}}}}=2a/m$ as given in the Supplementary Information. Similarly, for the 3D release energy, we use a parabolic fitting function $a{({t}_{{{{\rm{3D}}}}}+{t}_{{{{\rm{fix}}}}})}^{2}+b$ to our 3D-expansion data. Here, *t*_fix_ is the fixed 1D expansion time. We extract ${{{{\mathcal{E}}}}}_{{{{\rm{3D}}}}}$ from the fit via ${{{{\mathcal{E}}}}}_{{{{\rm{3D}}}}}=2a/m$.

### Atom loss and lifetime measurements

**Fig. 8:**
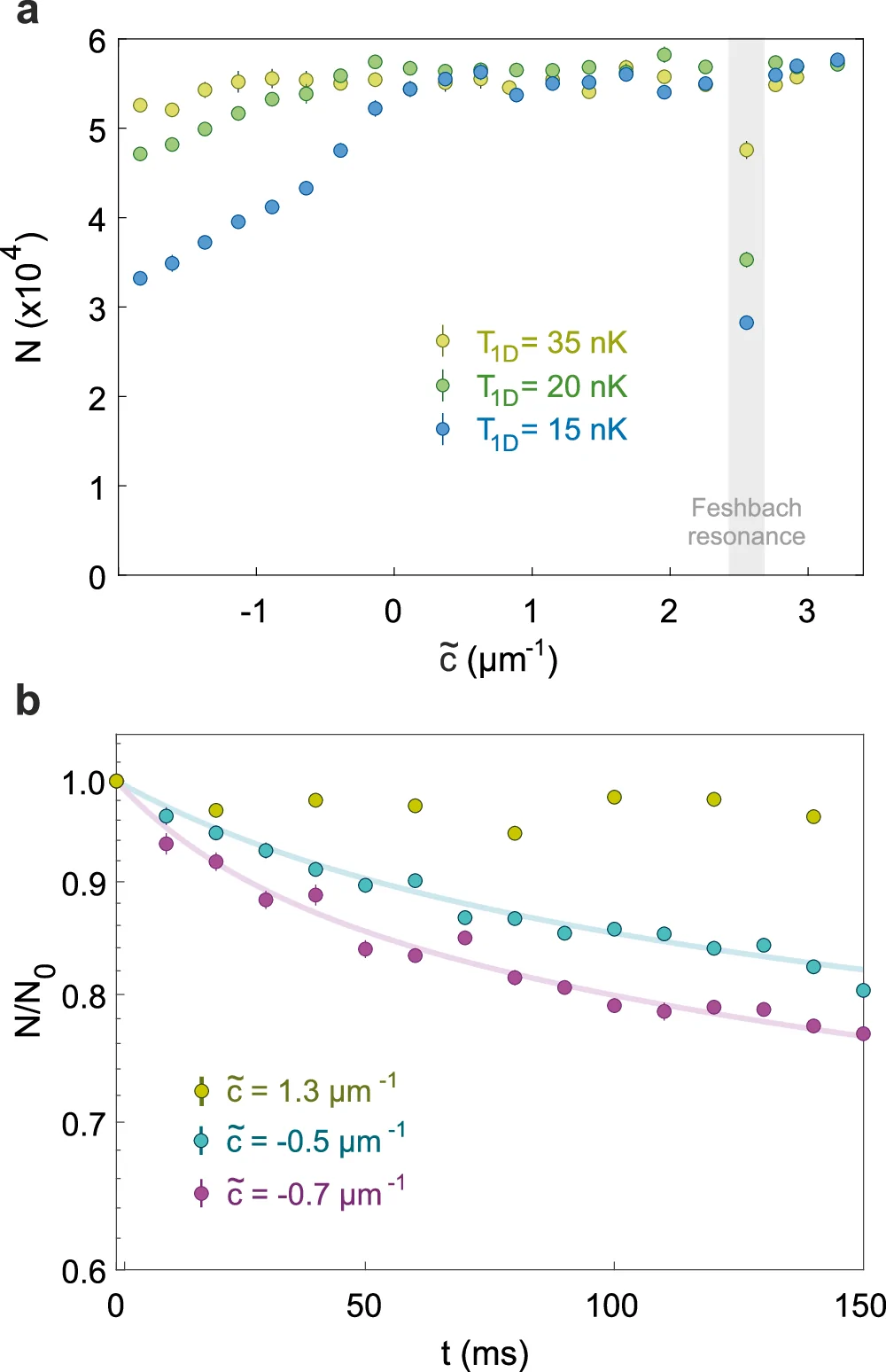
Atom loss for different temperatures and interaction strengths. **a** Number of atoms after the interactions ramp as a function of $\tilde{c}$ for different temperatures. Blue, green, and yellow correspond to 1D temperatures *T*_1D_ of 15 nK, 20 nK, 35 nK, respectively. A Feshbach resonance is indicated by a gray shaded region. **b** Evolution of normalized atom number for different values of $\tilde{c}$ at a temperature of 15 nK. Each data point is an average of five repetitions, and the displayed error is equal to the standard error. The solid curves are phenomenological fits to the data, assuming three- body losses with an adjustable global rate.

In our experiment, short-range molecular processes lead to atom loss. In Fig. 8a, we show the atom losses during the interaction ramp corresponding to the measurements given in Fig. 3b. In the repulsive regime $\tilde{c} > 0$, the atom number remains stable; we see enhanced losses for interactions below $\tilde{c}=0$. We attribute this atom loss primarily to the formation of bound states, which enhance inelastic scattering processes not taken into account by the 1D Hamiltonian Eq. (1). Since we use Cs atoms in the lowest hyperfine ground state $\left\vert F,{m}_{F}\right\rangle=\left\vert 3,3\right\rangle$, two-body inelastic processes are suppressed. Therefore, these losses in our experiment primarily originate from the three-body inelastic scattering. The stability of the gas depends both on the interaction strength and on the initial conditions of the protocol. The stronger the attraction, the tighter the bound states, with more frequent inelastic scattering. For a fixed $\tilde{c}$, the atom loss decreases at higher temperatures, as the probability of forming larger strings decreases at higher temperatures. While for *T*_1D_ = 20 and 35 nK, the losses are less than 20%, for the coldest *T*_1D_ = = 15 nK, we observe losses around 40% for the strongest attractive interaction. Even with such high losses, the agreement between experimental data for the momentum distribution after 1D expansion (normalized with atom number) and GHD results, as shown in Fig. 3a, remains very good. Next, we measure the lifetime of the gas for different interaction strengths. In Fig. 8b, we show the normalized atom number as a function of hold time *t* after the completion of the $\tilde{c}$ ramp for three different $\tilde{c}$ target values: $\tilde{c}=1.3,-0.5,-0.7$ μm^−1^. For these measurements, we set *t*_1D_ = 0 ms and *t*_3D_ = 36 ms. In the repulsive regime, we do not observe any significant atom loss, however, in the attractive regime, we observe around 20% atom loss over *t* = 150 ms. Here, the density of the gas increases, resulting in higher three-body losses. We fit the data with a phenomenological model for three-body losses, enhanced by the increased density due to string formation. Assuming the strings are dilute, losses are dominated by the process involving three particles belonging to the same string. To simplify the calculation, we will assume that, if a loss event occurs inside a string, the whole string is lost. This overestimates the losses, since it does not consider the possibility that long strings would break into shorter ones after the loss event. We therefore describe the three-body loss event as a one-body loss of the whole string object, with a phenomenological rate.

The typical rate equation describing three-body losses can therefore be expressed as

4 $$\frac{d{N}_{n}}{dt}=-{k}_{3}{P}_{n}{N}_{n}$$

where *N*_*n*_ is the number of strings of size *n*, *k*_3_ is a phenomenological three-body loss rate, and the quantity *P*_*n*_ expresses the probability to find three particles at the same position in the string. From dimensional analysis, and considering that *c* sets the size of strings, we expect the scaling *P*_*n*_ ∝ *c*^2^*n*(*n* − 1)(*n* − 2), where the *n* dependent factors express the different ways in which a triplet can be chosen inside the n-body cluster. Absorbing the proportionality factor into a redefinition of *k*_3_, we obtain the following time dependence for the string populations

5 $${N}_{n}(t)={N}_{n}(0){e}^{-{k}_{3}{c}^{2}n(n-1)(n-2)t}.$$

Assuming this time dependence and initial conditions predicted by our GHD modeling, we fit the total particle number as a function of time $N(t)={\sum}_{n}{N}_{n}(t)$ to the loss curves in Fig. 8b with *k*_3_ as the only free parameter. The agreement is remarkable, with a global fitted *k*_3_ = 1.10(3) × 10^−8^ cm^2^ s^−1^. For comparison, the loss rate in 3D in the vicinity of the magnetic field in which our experiment operates was measured to a value of ${k}_{3}^{\,{\mbox{(3D)}}\,}\approx 3\times 1{0}^{-27}$ cm^6^s^−1^^75^. We convert our fitted value into a 3D expectation, considering integration over the transverse wavefunction of the gas, resulting in the relation

6 $${k}_{3}^{\,{\mbox{(3D)}}}\simeq {k}_{3}3{\pi }^{2}{a}_{{\mbox{ho}}\,}^{4},$$

which is valid far away from the confinement-induced resonance. Using this, we find a value of 1.50(4) × 10^−27^ cm^6^ s^−1^ for the corresponding 3D loss rate predicted from the measured best fit value for our dataset. The agreement is remarkable, considering the approximations made. We conclude that the losses we observe in the experiment are well captured by our three-body loss model.

## Supplementary information

Supplementary Information

Transparent Peer Review file

## Data Availability

Experimental and simulation data are available on Zenodo^73^.
