# Supplementary material for: Probing Bethe strings in an attractive one-dimensional Bose gas
Source: Nat Commun. 2026 Aug 29;17:10330. doi: 10.1038/s41467-026-76018-0 (PMC13623881; doi:10.1038/s41467-026-76018-0)
Supplement: Supplementary file 1 [file 41467_2026_76018_MOESM1_ESM.pdf]

Supplementary Information of "Probing Bethe strings in an attractive one-dimensional Bose gas"

Milena Horvath,[1] Alvise Bastianello,[2, 3] Sudipta Dhar,[1] Rebekka Koch,[4] Yanliang Guo,[1] Jean-Sébastien Caux,[4] Manuele Landini,[1] and Hanns-Christoph Nägerl[1]

[1] *Institut für Experimentalphysik und Zentrum für Quantenphysik, Universität Innsbruck, Technikerstraße 25, Innsbruck, 6020, Austria*
[2] *Technical University of Munich, TUM School of Natural Sciences, Physics Department, 85748 Garching, Germany*
[3] *Munich Center for Quantum Science and Technology (MCQST), Schellingstr. 4, 80799 München, Germany*
[4] *Institute of Physics and Institute for Theoretical Physics, University of Amsterdam, PO Box 94485, 1090 GL Amsterdam, The Netherlands*

1. SUPPLEMENTARY NOTE 1: SUMMARY OF GENERALIZED HYDRODYNAMICS

Here we provide a compact summary of generalized hydrodynamics (GHD), summarizing the main equations and how theory describes the momentum measurement after one-dimensional (1D) expansion. A more detailed discussion for the interested reader can be found in Supplementary Note 4. The Hamiltonian of the Lieb-Liniger model in a trapping potential $V(z)$ is:

$$\hat{H}_{\rm LL} = -\frac{\hbar^2}{2m}\sum_i \frac{\partial^2}{\partial z_i^2} + \frac{\hbar^2 \tilde{c}}{2m}\sum_{i\neq j}\delta(z_i - z_j) + \sum_i V(z_i)\,. \tag{S1}$$

We assume this Hamiltonian provides a good description of the 1D tubes with trapping potential $V(z) = \frac{1}{2}m\omega_\parallel^2 z^2$. In the limit where the trap-induced inhomogeneity is sufficiently smooth, one can invoke a local-density approximation and locally treat the system as if it was integrable. This is the approach of GHD [1, 2], a non-perturbative kinetic theory for integrable systems, governed at the Euler scale by the main equation

$$\partial_t \rho_n(\lambda) + \partial_z[v_n^{\rm eff}(\lambda)\rho_n(\lambda)] + \partial_\lambda[F_n^{\rm eff}(\lambda)\rho_n(\lambda)] = 0\,. \tag{S2}$$

The root density $\rho_n(\lambda) \to \rho_{n;t,z}(\lambda)$ is the phase-space density of Bethe strings of species n in the position-rapidity plane. For $\tilde{c} > 0$, $\rho_{n=1}$ is the only non-zero root density, since no bound states are present. The effective velocity $v_n^{\rm eff}$ [1, 2] is renormalized by interactions accounting for non-trivial scattering, while the weak integrability-breaking induced by the trap $\partial_z V \neq 0$ [3] and the effect of slow interaction changes $\tilde{c} \to \tilde{c}(t)$ [4] are captured by the effective forces $F_n^{\rm eff}$. Reference [5] connects the GHD equations across $\tilde{c} = 0$ and determines the population of Bethe strings. Within GHD, the internal and potential energies of the gas are $U = \sum_n \int {\rm d}z{\rm d}\lambda\, E_n(\lambda)\rho_n(\lambda)$ and $E_V = \sum_n \int {\rm d}z{\rm d}\lambda\, V(z) n\rho_n(\lambda)$ respectively.

The combined 1D and 3D expansion of our protocol can be also described within GHD. After reaching the dilute regime upon expanding in 1D with $F_n^{\rm eff} = 0$, Bethe strings travel with their bare velocity $\hbar\lambda/m$. Then, the transverse trap is removed and interactions quenched to zero. Particles released from a Bethe string acquire new momenta p due to the converted binding energy, resuming the expansion and leading to a longitudinal density profile $d(z)$

$$d(z) = \sum_n \int {\rm d}\lambda{\rm d}p\,\delta\left(z - \tfrac{t_{\rm 1D}\hbar\lambda + t_{\rm 3D}p}{m}\right) C_n\left(\tfrac{p\hbar^{-1} - \lambda}{|\tilde{c}|}\right)\tfrac{n\bar{\rho}_n(\lambda)}{|\tilde{c}|}, \tag{S3}$$

where $\bar{\rho}_n(\lambda)$ is the rapidity distribution integrated over the whole cloud. C_n are universal bell-shaped function describing the momentum distribution obtained from an isolated Bethe string quenched to zero interaction, see Supplementary Note 4 for details. For simplicity, in Eq. (S3) we neglect the initial width of the cloud, and the short transient where the Bethe strings' velocity is renormalized by interactions. These effects are included simulating the 1D expansion with the GHD Eq. (S2). In the repulsive phase, $d(z)$ maps to the rapidity distribution since $C_1(x) = \delta(x)$. This identification is lost in the attractive phase in the presence of Bethe strings. One can explicitly compute $\int {\rm d}z\, x^2 C_n(x) = n(n^2-1)/12$, leading to a simple expression for the variance of the expanded density profile $\langle z^2\rangle \equiv \int {\rm d}z\, z^2 d(z) \Big/ \int {\rm d}z\, d(z)$

$$\langle z^2\rangle = \frac{2(t_{\rm 1D}+t_{\rm 3D})^2}{m}\langle E_{\rm com}\rangle + \frac{2t_{\rm 3D}^2}{m}\langle E_{\rm b}\rangle\,, \tag{S4}$$

which we use to extract the average center of mass and binding energies upon varying the 1D and 3D expansions. The average of $E_{\rm com}$ and $E_{\rm b}$ is taken over the root densities, normalized to the particles number.

The pair correlation integrated over the trap is formally defined as $G_2 \equiv \left\langle \sum_{i\neq j} \delta(z_i - z_j) \right\rangle$. In Supplementary Note 4, we give an explicit formula for G_2 in terms of the root densities Eq. (S12), and provide a more detailed overview of GHD and a discussion of finite expansion's time effects.

2. SUPPLEMENTARY NOTE 2: SIMULATING THE TONKS-GIRARDEAU REGIME

At strong repulsive interaction, the 1D Bose gas is well described by a gas of hard-core bosons, also known as Tonks-Girardeau (TG) regime and it is amenable of a straightforward theoretical treatment that we briefly recap [6]. Through a Jordan-Wigner transformation, hard-core bosons can be mapped into a system of free fermions. Hence, their hydrodynamics is that of free particles, satisfying the equation $\partial_t \rho_{t,z}(\lambda) + v(\lambda)\partial_z \rho_{t,z}(\lambda) - \partial_z V(z) \partial_k \rho_{t,z}(\lambda) = 0$, with $v(\lambda) = \partial_\lambda E(\lambda)$ and $E(\lambda) = \frac{\hbar^2 \lambda^2}{2m}$ and $\rho_{t,z}(\lambda)$ being the local rapidity distribution. The initial condition to the hydrodynamic equations is given by thermal states, which we compute within the local density approximation $\rho_{t=0,z}(\lambda) = \frac{1}{2\pi}\left(1 + e^{\beta[E(\lambda)-\mu+V(z)]}\right)^{-1}$, with the inverse temperature β and the chemical potential μ being fitting parameters.

We compute the momentum distribution within a local density approximation (LDA). We fix the position z, and consider a homogeneous state described by the rapidity distribution $\rho_{t,z}(\lambda)$. On homogeneous states, one defines the fermionic correlator $F_z(\Delta z) = \int {\rm d}\lambda\, e^{i\lambda\Delta z} \rho_{t,z}(\lambda)$, from which the bosonic correlation $g_{1,z}(\Delta z) \equiv \langle \hat{\psi}^\dagger(z + \Delta z/2)\hat{\psi}(z - \Delta z/2)\rangle$ can be computed, where we conveniently introduced the creation-annihilation bosonic fields $\hat{\psi}^\dagger(z)$ and $\hat{\psi}(z)$ obeying canonical commutation relations $[\hat{\psi}(z), \hat{\psi}^\dagger(z')] = \delta(z - z')$. $g_{1,z}(\Delta z)$ is found within LDA by solving certain Fredholm integral equations [7] (see also Ref. [8] for our notation)

$$g_{1,z}(\Delta z) = \det[1 - 2\mathcal{F}_{(\Delta z,0)}] \left[\mathcal{F}_{(\Delta z,0)} * (1 - 2\mathcal{F}_{(\Delta z,0)})^{-1}\right](\Delta z, 0)\,, \tag{S5}$$

where $\mathcal{F}_{(\Delta z,0)}$ is an operator acting on functions with support in $(\Delta z, 0)$ and with entries $\mathcal{F}_{(\Delta z,0)}(a, b) \equiv F_z(a - b)$. Its action on a test function f is defines as $[\mathcal{F}_{b\in(\Delta z,0)}](a) \equiv \int_{(\Delta z,0)} {\rm d}b\, \mathcal{F}_{(\Delta z,0)}(a, b) f(b)$. For brevity, we define the operator product on this space as $[A * B](a, b) \equiv \int_{y\in(\Delta z,0)} {\rm d}y\, A(a, y)B(y, b)$. In Eq. (S5), after having computed the operator $\left[\mathcal{F}_{(\Delta z,0)} * (1 - 2\mathcal{F}_{(\Delta z,0)})^{-1}\right]$, one has to focus on its entries at the edge of the definition domain $\left[\mathcal{F}_{(\Delta z,0)} * (1 - 2\mathcal{F}_{(\Delta z,0)})^{-1}\right](\Delta z, 0)$

In practice, determinants and matrix inversions are computed by discretizing the operator on a finite grid (about 120 points in our simulations). Finally, the momentum distribution is obtained by Fourier transform. More specifically, we define the LDA position-dependent momentum distribution as

$$P_z(p) = \int \frac{{\rm d}\Delta z}{2\pi}\, e^{-ip\Delta z} g_{1,z}(\Delta z)\,. \tag{S6}$$

$P_z(p)$ describes the probability distribution in the momentum-space (p, z) phase space in the longitudinal direction obtained right after the release of the three dimensional optical lattice. The final comparison with experimental data is obtained by further propagating the particles for a finite three dimensional TOF, assuming that $P_z(p)$ describes the number of particles starting at z and with velocity $v(p) = \hbar p/m$.

3. SUPPLEMENTARY NOTE 3: CHARACTERIZING THE RAPIDITY DISTRIBUTION IN THE TG REGIME

Theory predicts the longitudinal density profile of the expanded cloud after the system has entered a sufficiently dilute regime. Therefore, benchmarking the validity of this approximation is of key importance. In the repulsive regime, the momentum distribution of a dilute gas is approximated by the rapidity distribution, but at finite density, interactions spoil this identification. Computing the momentum distribution at finite density and for arbitrary interaction strength is theoretically challenging, but it is accessible in the TG regime (see Supplementary Note 2). We use this to benchmark our experimental results. We characterize our rapidity measurement protocol by first matching the experiment with the simulation in the TG regime for different 1D expansion times $t_{\rm 1D}$. In our experiment, after lattice loading, we ramp $a_{\rm 3D}$ from 210 a_0 to approximately 750 a_0 in 150 ms. We then apply a 808-nm anti-trapping beam that flattens out the longitudinal harmonic trap. We estimate that the longitudinal confinement of the tubes is

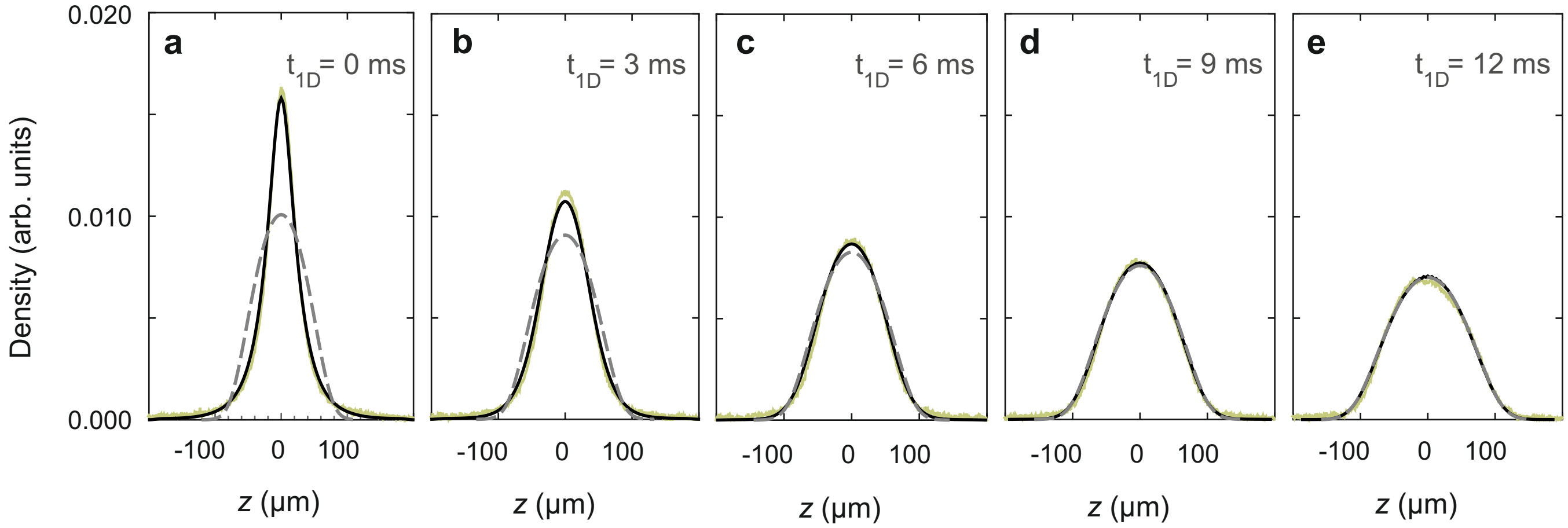

Supplementary Fig. 1. **1D expansion in TG regime.** **a** to **e**, TOF distributions of a TG gas following an expansion in 1D, with $t_{1\mathrm{D}} = 0$ to 12 ms. z denotes the spatial position after TOF. The experimental data and simulation results are plotted in green, and black respectively. The black curve is the density profile obtained expanding the true momentum distribution, whereas the grey dashed curve is obtained approximating the momentum distribution with the rapidity distribution. Each experimental data is the average of five repetitions.

flattened for a region of approximately 80 μm around the center of the trap without affecting the transverse trapping. We allow the gas to expand in 1D for variable time $t_{1\mathrm{D}}$, before taking a standard time-of-flight (TOF) absorption image. In Supplementary Fig. 1, we show the TOF distribution of the gas after expansion in 1D for $t_{1\mathrm{D}} = 0$ to 12 ms. Throughout the 1D expansion, we see good agreement with the simulation results. For $t_{1\mathrm{D}} = 0$ ms, the momentum and rapidity distributions are strikingly different. As $t_{1\mathrm{D}}$ increases, the distributions broaden and approach the rapidity distribution. After $t_{1\mathrm{D}} = 6$ ms, the TOF distributions coincide with the rapidity distribution of the gas.

4. SUPPLEMENTARY NOTE 4: GHD AND SIMULATIONS AT FINITE INTERACTIONS

To give quantitative theoretical predictions for the finite interactions, we employ thermodynamic Bethe ansatz (TBA) [9] to determine the initial state, and GHD to follow the time evolution. Throughout this section, we use for simplicity adimensional units rescaling coordinates z_i by a unit length $\ell = 1$ μm, in such a way the Bose gas Hamiltonian reads

$$\hat{H} = -\sum_i \frac{\partial^2}{\partial z_i^2} + c \sum_{i \neq j} \delta(z_i - z_j) + \sum_i V(z_i)\,, \tag{S7}$$

with $c = \tilde{c}\,\ell$.

1. Summary of notation

It is convenient to define a unified notation. In integrable models, interactions are captured by "dressing" bare quantities. For an arbitrary test function $\tau_n(\lambda)$, the dressing operation $\tau_n(\lambda) \to \tau_n^{\mathrm{dr}}(\lambda)$ is defined as the solution of the integral equation

$$\tau_n^{\mathrm{dr}}(\lambda) = \tau_n(\lambda) - \sum_{n'} \int \frac{\mathrm{d}\lambda'}{2\pi} \varphi_{n,n'}(\lambda - \lambda') \vartheta_{n'}(\lambda') \tau_{n'}^{\mathrm{dr}}(\lambda')\,. \tag{S8}$$

Above, the integral over the rapidities is on the whole real axis, and the summation over the internal index n runs over the domain discussed below. In Eq. (S8) one defines the filling fraction $\vartheta_n(\lambda) \equiv \rho_n(\lambda)/(2\pi(\partial_\lambda p_n(\lambda))^{\mathrm{dr}}$, with $p_n(\lambda)$ the bare momentum of the quasiparticle and $\varphi_{n,n'}(\lambda)$ the interaction-dependent scattering kernel. In the repulsive phase $c > 0$ one has only terms for $n = 1$, and $p_{n=1}(\lambda) = \lambda$, $\varphi_{n=1,n'=1}(\lambda) = -\frac{2c}{\lambda^2 + c^2}$ and bare energy $E_{n=1}(\lambda) = \lambda^2$.

In contrast, in the attractive case $n \in \mathbb{N}$ and one has $p_n(\lambda) = n\lambda$, $E_n(\lambda) = n\lambda^2 - \frac{c^2}{12}n(n^2-1)$ and scattering kernel $\varphi_{n,n'}(\lambda) = (1-\delta_{n,n'})a_{|n-n'|}(\lambda) + 2a_{|n-n'|+2}(\lambda) + 2a_{|n-n'|+4}(\lambda)... + 2a_{n+n'-2}(\lambda) + a_{n+n'}(\lambda)$, with $a_j(\lambda) = -\frac{4jc}{c^2j^2+4\lambda^2}$. The thermodynamics and hydrodynamics of integrable models can be expressed in terms of these functions.

2. Thermodynamics

The initial conditions of our experimental protocol within the repulsive phase are well-approximated by a thermal ensemble. Within the local density approximation, the filling function $\vartheta_{t=0,x}(\lambda)$ (where we suppress the $n-$label, since we focus on the repulsive phase $n=1$) is obtained by solving the following integral equations from thermodynamic Bethe ansatz [9]

$$\log[1/\vartheta_{t=0,z}(\lambda) - 1] = \beta[E(\lambda) - \mu_\parallel + V(z)] - \int \frac{\mathrm{d}\lambda'}{2\pi}\varphi(\lambda-\lambda')\log[1-\vartheta_{t=0,z}(\lambda')]\,. \tag{S9}$$

When estimating the tube population in Supplementary Note **??**, the chemical potential of each tube is renormalized by the transverse potential $\mu_\parallel \to \mu - V_\perp(x,y)$, where $V_\perp(x,y)$ is the transverse trapping potential in the $x-y$ plane. The global chemical potential μ and global inverse temperature β are then considered fitting parameters.

3. Hydrodynamics

We simulate the evolution with GHD, expressed in the space of filling fractions [1, 2]

$$\partial_t\vartheta_n + v_n^{\mathrm{eff}}\partial_x\vartheta_n + F_n^{\mathrm{eff}}\partial_\lambda\vartheta_n = 0 \tag{S10}$$

and equivalent to Eq. (S2), where we omitted all the variables for compactness. The GHD equations in the fillings' space are more stable for numerical purposes. The effective velocity can be computed as $v_n^{\mathrm{eff}}(\lambda) = (\partial_\lambda E_n)^{\mathrm{dr}}/(\partial_\lambda p_n)^{\mathrm{dr}}$, while the effective force $F_n^{\mathrm{eff}}(\lambda) = \mathcal{F}_n^{\mathrm{dr}}(\lambda)/(\partial_\lambda p_n)^{\mathrm{dr}}$ combines the effect of the trap, and of interaction changes in time $\mathcal{F}_n(\lambda) = -n\partial_z V + \partial_t c \sum_{n'}\int \frac{\mathrm{d}\lambda'}{2\pi}\partial_c\Phi_{n,n'}(\lambda-\lambda')(\partial_{\lambda'}p_{n'})^{\mathrm{dr}}\vartheta_{n'}(\lambda')$, where one defines $\Phi_{n,n'}(\lambda) = \int^\lambda \mathrm{d}\lambda'\,\varphi_{n,n'}(\lambda')$. The above GHD equations describe the hydrodynamic evolution within the repulsive or attractive phase separately, but for passing from one to another the proper boundary conditions are needed. Physically, they describe how particles form Bethe strings by passing from the repulsive to the attractive phase. Using a maximum-entropy argument valid in the regime of slow interaction changes, these equations have been derived in Refs. [5, 10]

$$\vartheta_{n;z}(\lambda)\Big|_{c\to 0^-} = 1 - \frac{\sinh(n\Omega_z(\lambda)/2)\sinh((n+2)\Omega_z(\lambda)/2)}{\sinh^2((n+1)\Omega_z(\lambda)/2)} \tag{S11}$$

with $\Omega_z(\lambda)$ defined from the repulsive root density at vanishing interactions $\Omega_z(\lambda) = \lim_{c\to 0^+}\log\left[1 + 1/(2\pi\rho_{n=1;z}(\lambda))\right]$. During the evolution, the density and energy profiles are computed in local density approximation. An analytical expression for the local pair correlator has been obtained through the Hellmann-Feynmann theorem [4, 11]

$$\langle[\hat\psi^\dagger(z)]^2[\hat\psi(z)]^2\rangle = \sum_n \int \mathrm{d}\lambda\left\{\partial_c E_n(\lambda)\rho_n(\lambda) + \frac{1}{2\pi}\partial_\lambda E_n(\lambda)\vartheta_n(\lambda)f_n^{\mathrm{dr}}(\lambda)\right\}, \tag{S12}$$

whereas the total integrated pair correlation is $G_2 \equiv \int \mathrm{d}z\,\langle[\hat\psi^\dagger(z)]^2[\hat\psi(z)]^2\rangle$. Above, $f_n(\lambda) = \sum_{n'}\int \mathrm{d}\lambda'\,\Phi_{n,n'}(\lambda-\lambda')\rho_{n'}(\lambda)$, where, using a local density approximation, the TBA expressions are evaluated on the root density in position z.

4. The momentum distribution after the one-dimensional asymptotic expansion

The analytical computation of the momentum-distribution of a dense Bose gas is a formidable challenge. However, in the approximation of a dilute gas obtained after a 1D expansion from a dense gas, analytical results can be obtained.

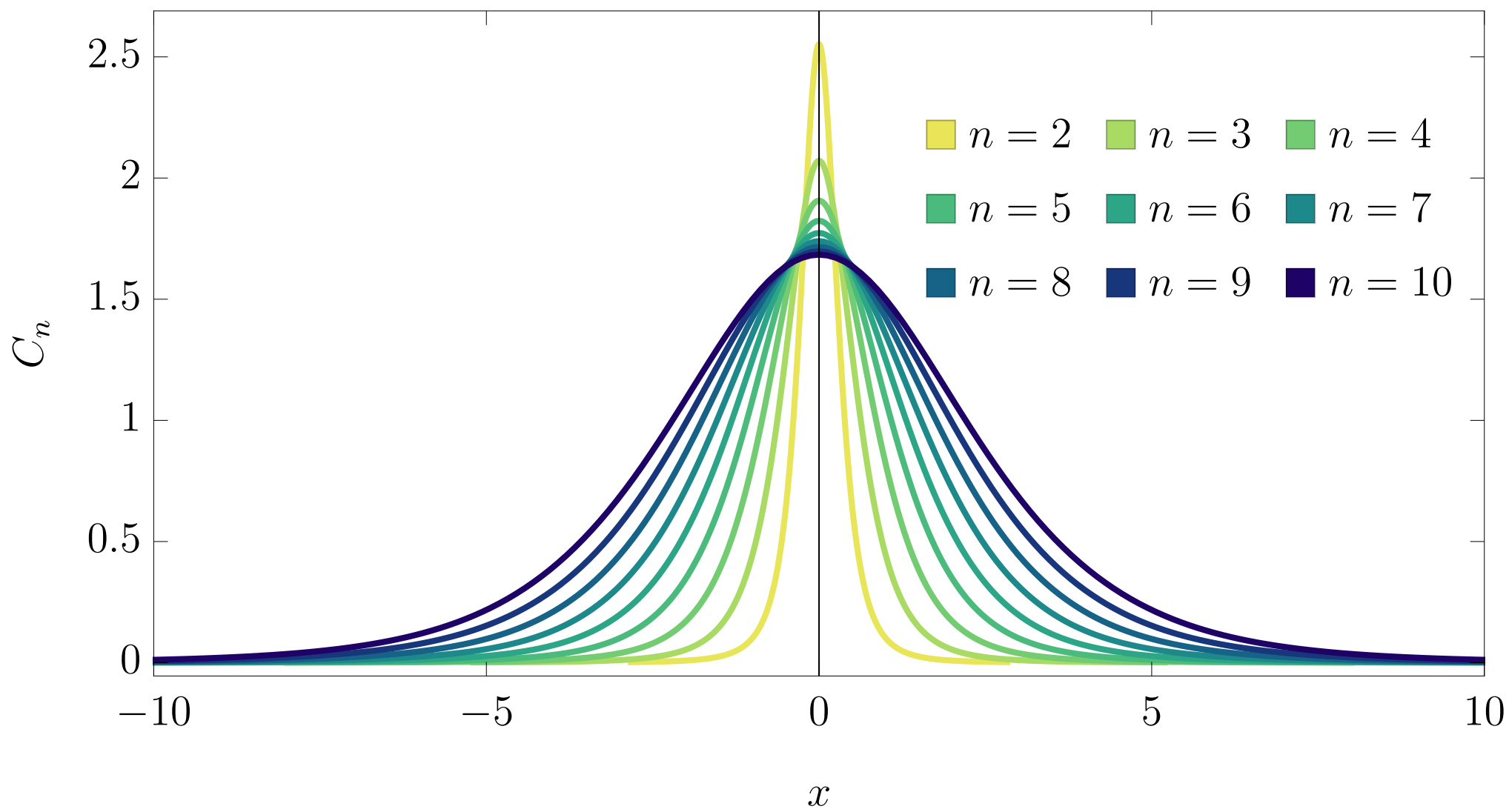

Supplementary Fig. 2. **The universal functions C_n.** We show the first universal functions appearing in the string-resolved momentum distribution after 1D expansion, see Eq. (S13) and related discussion.

In the repulsive case, isolated particles are well separated and the spatially resolved momentum distribution $P_z(p)$ coincides with the rapidity distribution $P_z(p) \simeq \rho_z(p)$ [12]. To compare with the TOF experimental measurements, we assume that when the three dimensional optical trap is switched off, $P_z(p)$ is the density distribution of particles in position z and with momentum p, which further freely evolve during the three-dimensional expansion.

In the attractive regime, Bethe strings complicate the picture. We assume Bethe strings are spatially well-separated, and they independently contribute to the final momentum distribution. The momentum distribution of a single Bethe string is given by the modulus square of the overlap between the Bethe string's wavefunction and plane waves. A Bethe string of rapidity λ and n particles has wavefunction $\psi_n(z_1 < z_2 < \cdots < z_n) \propto e^{i\sum_{j=1}^{n}(\lambda + i\frac{c}{2}(n+1-2j)z_j}$, and the wavefunction is symmetrically extended to other orderings of the coordinates. Taking advantage of Galilean invariance and the scaling with the interaction c, we can write the total spatially-resolved momentum distribution as

$$P_z(p) = \sum_{n=1}^{\infty} \int \mathrm{d}\lambda \frac{1}{|c|} C_n\left(\frac{p-\lambda}{|c|}\right) \rho_{n;z}(\lambda)\,, \tag{S13}$$

where the function C_n captures the contribution of each string, and it is defined as the modulus squared of the overlap of the Bethe string wavefunction with zero real rapidity and computed at unit interaction $c = -1$, which we call $\bar{\psi}_n$

$$C_n(x) = \int \mathrm{d}z_1 e^{-ixz_1} \int \mathrm{d}z_{j>1}\, \bar{\psi}_n^*(0, z_2, ..., z_n)\bar{\psi}_n(z_1, z_2, ..., z_n)\,. \tag{S14}$$

Notice that above there is no restriction on the order of the coordinates. This integral can be simplified with some tedious combinatorics which can be carried over on a laptop and we overview below. Modulus getting a factor $(n-1)!$, we can assume $z_2 < z_3 < ... < z_n$ and the non-trivial permuations are now z_1 and 0 with respect to the other coordinates. We expand the integral by summing over these domains. In the end, everything requires computing integrals in the form $I_j[q_1, ..., q_j] = \int_{y_1<...<y_j<\infty} \mathrm{d}^n y\, e^{i\sum_{a=1}^{j} q_j y_j}$ with imaginary q_j. By explicitly integrating the rightmost coordinate y_j, a simple recursive equation is found $I_j[q_1, ..., q_{j-1}, q_j] = \frac{i}{q_j} I_{j-1}[q_1, ..., q_{j-1} + q_j]$, which eventually gives a quick tabulation of $C_n(z)$. A Mathematica commented notebook that tabulates C_n is provided on Zenodo [13]. In Supplementary Fig. 2 we show the first universal functions $C_n(z)$. They are bell-shaped functions normalized to the number of particles in the Bethe string $\int \mathrm{d}z\, C_n(z) = n$ and of increasing width for larger n.

While the full profile for $C_n(z)$ is tedious to recover analytically for large n, the variance is rather easy to compute. We consider directly the momentum variance $\langle p^2 \rangle_{n,\lambda}$ of a Bethe string of rapidity λ and n components. We can leverage on the knowledge of the energy of this state $E_n(\lambda) = \langle H \rangle_{n,\lambda} = n\lambda^2 - \frac{c^2}{12} n(n^2-1)$. From the Hellmann-Feynman theorem we know $\partial_c E_n(\lambda) = \langle \partial_c H \rangle_{n,\lambda}$, and with the explicit observation that $\partial_c H = \sum_{i \neq j} \delta(z_i - z_j)$ we

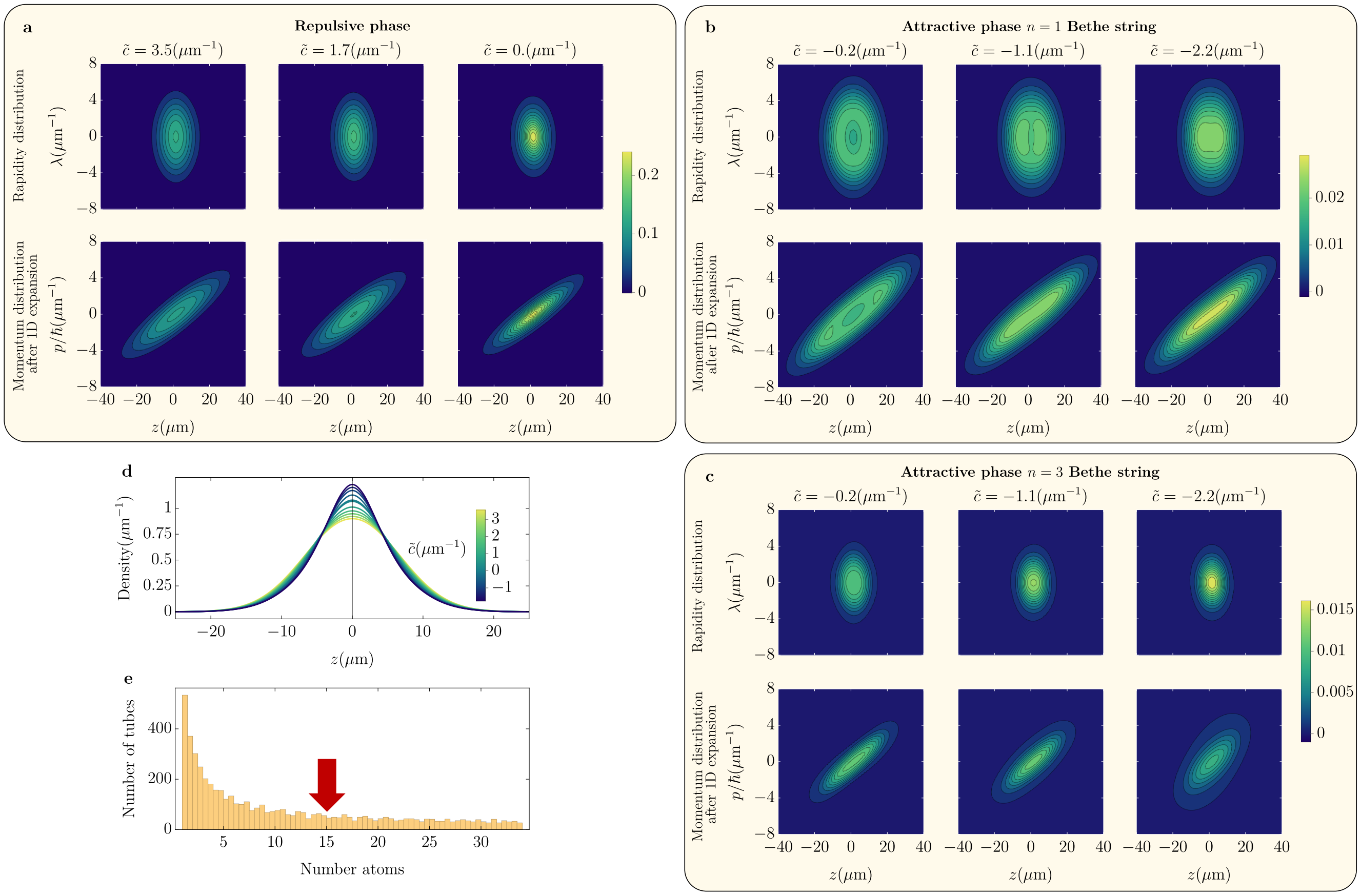

Supplementary Fig. 3. **Example of a typical evolution with GHD.** GHD evolution of a representative tube of 15 atoms in a harmonic trap with frequency 27.6 Hz, initially described by a thermal ensemble with temperature $T_{1D} = 20$ nK. **a** to **c**, phase space density of the rapidity (first row) and momentum distribution per particle after 1D expansion of 10ms (second row). **a** shows the repulsive phase at different interactions, **b** to **c** focus on the example of the $n = 1$ and $n = 3$ Bethe strings. **d**, density profile in the harmonic trap, for different interactions. **e**, estimated atom number populations in the 1D tubes for a crossover temperature of 10 nK.

observe

$$\langle p^2 \rangle_{n,\lambda} = \langle H - c \sum_{i,j} \delta(x_i - x_j) \rangle_{n,\lambda} = E_n(\lambda) - c\partial_c E_n(\lambda) = n\lambda^2 + \frac{c^2}{12} n(n^2-1)\,. \tag{S15}$$

Specifying $\lambda = 0$ and $c = -1$, we connect with the functions C_n getting $\int \mathrm{d}y\, C_n(y) = \frac{1}{12} n(n^2-1)$. The total variance of the momentum distribution after 1D expansion is obtained by summing over all the strings with the proper weight $\rho_n(\lambda)$.

5. Numerical discretization

The filling functions appearing in the GHD Eqs. (S10) are discretized on a finite grid in the rapidity and real space. The main bottleneck for the simulations is the solution of the (discretized) integral equations defining the dressing operation, whose matrices grow with the number of strings times the number of points in the rapidity discretization. We use 100 points in the space discretization, 50 points in the rapidity's grid for a maximum number of 25 strings for Fig. 3, and increase it to 30 strings for Fig. 4 where we did not numerically expanded in 1D, but directly computed the energies from the GHD. For the time-evolution of GHD, we use the method of characteristics with the second order implementation described in Ref. [4]. In Supplementary Fig. 3, we provide further details on a typical simulation

focusing on theory only. For the sake of concreteness, we focus on one of the datasets discussed in Fig. 3, more precisely we consider the case $T_{1D} = 20$ nK, and we focus on a single representative tube with 15 atoms. In Supplementary Fig. 3**a** to **c**, we show density plots of the rapidity distribution in the harmonic trap, and the spatially-resolved momentum distribution after 1D expansion of 10 ms, for different interaction strengths. Supplementary Fig. 3**a** shows the repulsive side (no strings), whereas in Supplementary Fig. 3**b** and **c**, we consider the attractive phase and focus, as an example, on the $n = 1$ Bethe string (one particle) and $n = 3$ Bethe string (three-particle bound state) respectively. As the interaction $\tilde{c}$ is changed from strongly to weakly repulsive, and then from weakly attractive to strongly attractive the rapidity distribution gets squeezed in the center of the trap. Upon 1D expansion, the momentum distribution in the repulsive branch and of the $n = 1$ Bethe string are simply a deformation of the rapidity distribution obtained ballistically propagating the particles in the z-direction, the magnitude of the velocity increases with the momentum giving the apparent rotation of the oval. For the $n = 3$ Bethe string, this effect is superimposed with the energy release upon breaking the bound states, that dilates the distribution in the vertical direction. This effect is stronger at larger values of attractive interaction. In Supplementary Fig. 3**d**, we show the density profile within the harmonic trap, where the squeezing in the center of the trap is evident. Finally, in Supplementary Fig. 3**e** we provide the estimated atom population across the tubes (see Methods). The dimensional-crossover temperature that best matches experimental data is 10 nK. For completeness, in Supplementary Fig. 4 we show a full comparison of the experimental expanded cloud with results of GHD simulations for the dataset corresponding to $T_{1D} = 15$ nK and $T_{1D} = 35$ nK shown in Fig. 3.

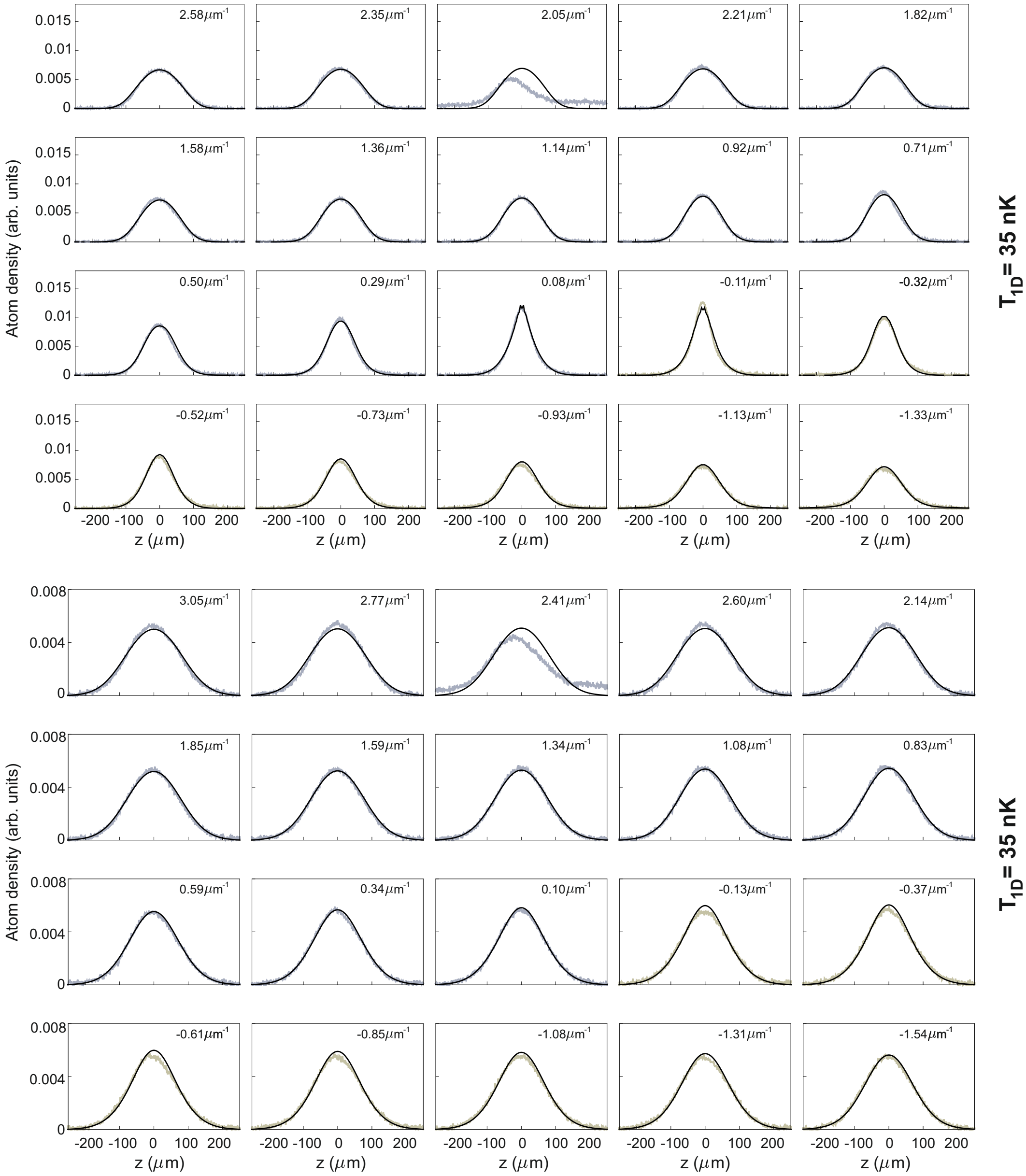

Supplementary Fig. 4. **Comparison of experimental data with GHD.** For completeness, we show the full dataset of experimental data compared with the results of GHD simulations, for the dataset of Fig. 3 corresponding to $T_{\rm 1D} = 15$ nK (top) and $T_{\rm 1D} = 35$ nK (bottom). See caption of Fig. 3 for details. We show the atom density after a longitudinal expansion of $t_{\rm 1D} = 10$ ms and $t_{\rm 3D} = 46.3$ ms in real space. Black solid lines are GHD curves, whereas colored lines are experimental data (repulsive: light blue, attractive: light green). The interaction strength $\tilde{c}$ is indicated on the right corner of each figure. The starting point of the interaction ramp is at $\tilde{c} = 2.58\,\mu{\rm m}^{-1}$, which is then linearly changed in time. The discrepancy shown at $\tilde{c} = 2.05\,\mu{\rm m}^{-1}$ is attributed to the fact that it is close to a Feshbach resonance. When ramping the magnetic field to reach less repulsive interactions and the attractive phase, we jump across the resonance to obtain a smooth interaction ramp. The small dents shown by GHD curves at the weakest repulsive and attractive interactions are numerical artifacts arising during the extraction of the 1D-expanded profile from the filling function. This can be reduced by improving the discretization. As shown in Fig. 3 of the main text, the effect of the changing interactions is more prominent for lower temperatures, in particular due to the increased Bethe strings' production upon entering in the attractive phase. Albeit still good, the agreement of the theoretical modeling with experimental data is worse in the hotter sample. This difference is arguably due to more important corrections to the modeling of the initial state, assuming thermally-distributed tubes with an uniform temperature. See also Supplementary Note **??**.

5. SUPPLEMENTARY NOTE 5: THE VIRIAL THEOREM AND GENERALIZED HYDRODYNAMICS

The virial theorem

$$2U - 2E_V - E_{\rm int} = 0 \tag{S16}$$

is well-known for equilibrium states [14]. In this section, we prove its validity for non-equilibrium states that are stationary with respect to the GHD equations. We work in the adimensional units of Supplementary Note 4 and start by manipulating the TBA expression for the pair correlator Eq. (S12). We consider the attractive phase, but the same calculations hold in the repulsive case considering the $n = 1$ case only. In the Lieb-Liniger model, the scattering kernel is not an independent function of λ and c, but a scaling function of their ratio $\partial_c \Phi_{n,n'}(\lambda) = -\frac{\lambda}{c}\partial_\lambda \Phi_{n,n'}(\lambda) = -\frac{\lambda}{c}\varphi_{n,n'}(\lambda)$. Using this fact, and the symmetry of the kernel and of the dressing function $\sum_n \int {\rm d}\lambda \vartheta_n(\lambda) a_n(\lambda) b_n^{\rm dr}(\lambda) = \sum_n \int {\rm d}\lambda \vartheta_n(\lambda) a_n^{\rm dr}(\lambda) b_n(\lambda)$ for any function $a_n(\lambda)$ and $b_n(\lambda)$, with straightforward manipulations we can write

$$\langle [\hat\psi^\dagger(z)]^2[\hat\psi(z)]^2\rangle = \frac{1}{c}\sum_n \int {\rm d}\lambda\, \left[\partial_c E_n(\lambda) + \lambda\partial_\lambda E_n(\lambda)\right]\rho_n(\lambda) + \frac{1}{c}\sum_n \int {\rm d}\lambda\, \lambda\partial_\lambda p_n(\lambda)(\partial_\lambda E_n)^{\rm dr}\vartheta_n(\lambda)\,. \tag{S17}$$

We now use the explicit expressions for the energy and momentum in the Lieb-Liniger model, and notice $\partial_c E_n(\lambda) + \lambda\partial_\lambda E_n(\lambda) = 2E_n(\lambda)$ and $\lambda\partial_\lambda p_n(\lambda) = p_n(\lambda)$. Multiplying both sides by c and integrating over z, we recognize the total interaction energy $E_{\rm int} = c\int {\rm d}z\, \langle[\hat\psi^\dagger(z)]^2[\hat\psi(z)]^2\rangle$ and total internal energy $U = \int {\rm d}z \sum_n \int {\rm d}\lambda\, E_n(\lambda)\rho_n(\lambda)$, where the $z-$dependence of the root density and filling fraction is left implicit for the sake of notation.

$$E_{\rm int} = 2U + \int {\rm d}z \sum_n \int {\rm d}\lambda\, p_n(\lambda)(\partial_\lambda E_n)^{\rm dr}\vartheta_n(\lambda)\,. \tag{S18}$$

So far we have not used the GHD equations yet, but only the local density approximation and symmetries of the TBA. The last step uses the GHD equations to further manipulate the remaining integral in Eq. (S18). In particular,

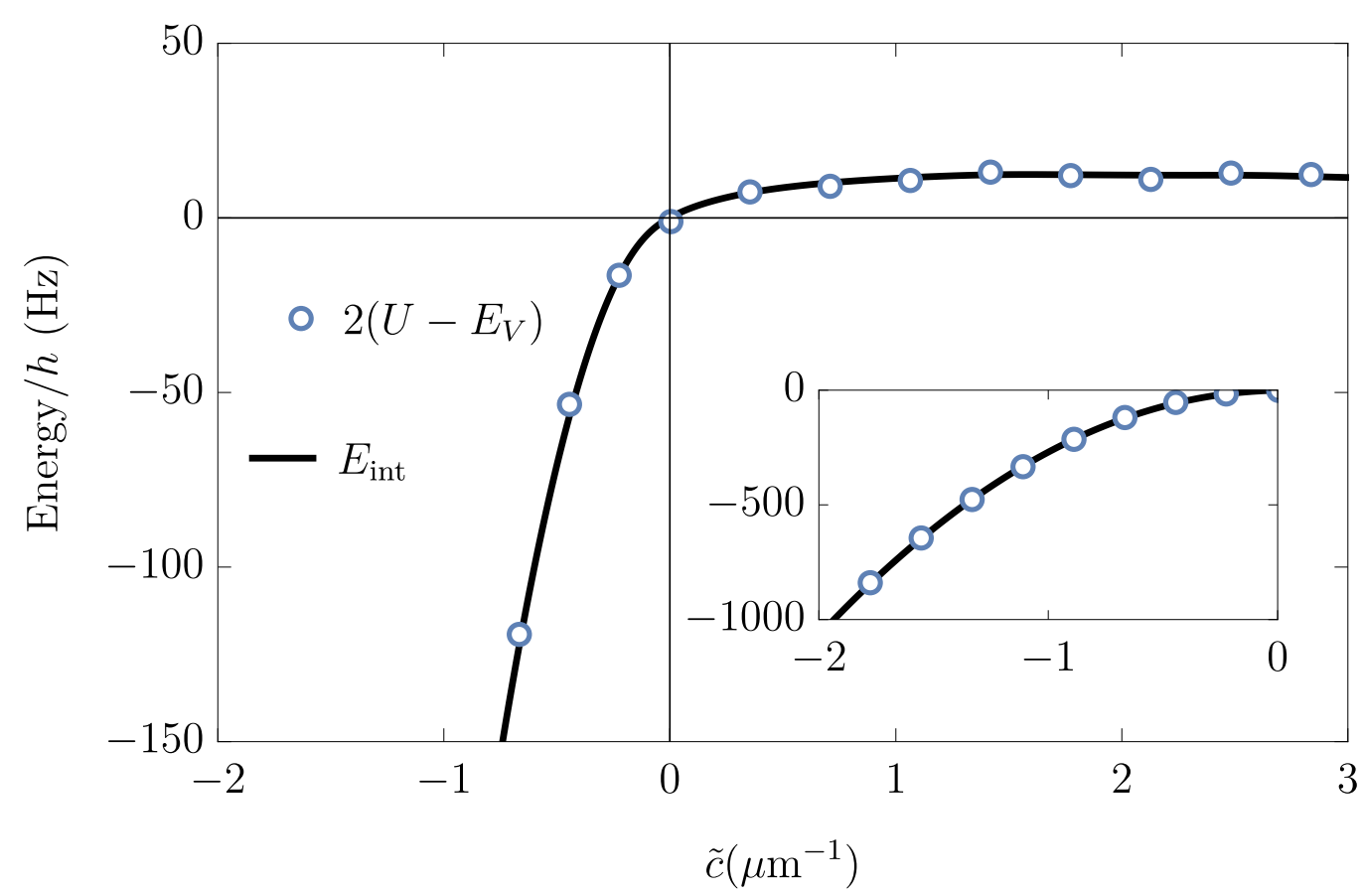

Supplementary Fig. 5. **Benchmark of the virial theorem in GHD simulations.** For the same dataset provided in Fig. 4, but focusing on theory only, we check that the corrections to the virial theorem due to the non-stationary nature of the state and to the interaction changes, as discussed in Eq. (S19), are negligible. The black line shows the total interaction energy obtained in the local density approximation from Eq. (S18). Markers are twice the difference between the internal and potential energies, and agree well with the interaction energy. The small oscillations shown in the repulsive phase are due to the corrections discussed in Eq. (S19), and are negligible compared to the experimental uncertainty. In the inset, we focus on the strongly attractive regime, the axis of the inset are same of the main plot, but on a different scale. In this regime, the binding energy of Bethe strings dominates the virial theorem. In these simulations, we considered 20 strings, in contrast with Fig. 4 where 30 strings have been used. Since the virial theorem holds for any filling fraction stationary to the GHD equations, the truncation in the number of strings does not spoil the agreement between the two curves.

it is possible to show

$$\int \mathrm{d}z \sum_n \int \mathrm{d}\lambda\, p_n(\lambda)(\partial_\lambda E_n)^{\mathrm{dr}}\vartheta_n(\lambda) = -\langle z\partial_z V\rangle - \frac{\mathrm{d}}{\mathrm{d}t}\left(\sum_n \int \mathrm{d}z \int \mathrm{d}\lambda\, z p_n(\lambda)\rho_n(\lambda)\right) + \\ + \partial_t c \int \mathrm{d}z \sum_n \int \frac{\mathrm{d}\lambda}{2\pi} z n \left(\sum_{n'} \int \mathrm{d}\lambda' \partial_c \Phi_{n,n'}(\lambda-\lambda')\rho_{n'}(\lambda')\right). \quad \text{(S19)}$$

The proof is technical and reported below. If the potential $V(z)$ is harmonic, then $z\partial_z V(z) = 2V(z)$ and one recovers the potential energy $\langle z\partial_z V\rangle = 2E_V$. In Eq. (S19), the total derivative is reminiscent of the virial theorem for classical particles, and there is a further contribution coming from interaction changes. If we consider stationary states, then $\partial_t c = 0$ and the total derivative vanishes as well, thus we recover the virial theorem $E_{\mathrm{int}} - 2U + 2E_V = 0$. The virial theorem remains a good approximation for slowly evolving states. In our simulations, we explicitly checked that the interaction energy computed through the virial theorem agrees with the internal energy from Eq. (S12), see Supplementary Fig. 5, and both agree well with the experimental data as shown in Fig. 4.

1. Proof of Eq. (S19)

To prove Eq. (S19), we first go through a convenient change of variables in the GHD equations. More precisely, we move from the (z, λ) plane to a pair of conjugated canonical variables $(z, \mathbf{p})$. We define

$$\mathbf{p}_n(\lambda) = p_n(\lambda) - \sum_{n'} \int \frac{\mathrm{d}\lambda'}{2\pi}\Phi_{n,n'}(\lambda-\lambda')\vartheta_{n'}(\lambda')(\partial_{\lambda'} p_{n'}(\lambda'))^{\mathrm{dr}} \quad \text{(S20)}$$

$$\mathbf{e}_n(\lambda) = E_n(\lambda) + nV(z) - \sum_{n'} \int \frac{\mathrm{d}\lambda'}{2\pi}\Phi_{n,n'}(\lambda-\lambda')\vartheta_{n'}(\lambda')(\partial_{\lambda'} E_{n'}(\lambda'))^{\mathrm{dr}} \quad \text{(S21)}$$

And consider the string-dependent change of variable $\lambda \to \mathbf{p} = \mathbf{p}_n(\lambda)$, likewise we define the filling fraction in the new space $\bar{\vartheta}_{n,z}(\mathbf{p})$ through the identity $\bar{\vartheta}_{n,z}(\mathbf{p}_n(\lambda)) = \vartheta_n(\lambda)$. We furthermore define $\mathcal{H}_n(z, \mathbf{p})$ as $\mathcal{H}_n(z, \mathbf{p}_n(\lambda)) = \mathbf{e}_n(\lambda)$, where the $z-$dependence is implicit in the integral Eq. (S21).

We now rewrite the GHD equations in the filling fraction space Eq. (S10) in terms of the new variables. Notice that $\partial_\lambda \mathbf{p}_n(\lambda) = (\partial_\lambda p_n)^{\mathrm{dr}}$ and $\partial_\lambda \mathbf{e}_n(\lambda) = (\partial_\lambda E_n)^{\mathrm{dr}}$, therefore $\frac{1}{(\partial_\lambda p_j)^{\mathrm{dr}}}\partial_\lambda \to \partial_{\mathbf{p}}$ and $v_n^{\mathrm{eff}}(\lambda) \to \partial_{\mathbf{p}}\mathcal{H}_n(x, \mathbf{p})$. Using these identities in Eq. (S10) one obtains $(\partial_t + \partial_t \mathbf{p}_j \partial_{\mathbf{p}})\bar{\vartheta}_n(\mathbf{p}) + \partial_{\mathbf{p}}\mathcal{H}_n \partial_x \bar{\vartheta}_n(\mathbf{p}) + \mathcal{F}^{\mathrm{dr}}\partial_{\mathbf{p}}\bar{\vartheta}_n(\mathbf{p}) = 0$, where it must be stressed that the change of variable $\lambda \to \mathbf{p}$ is time dependent since Eq. (S20) depends on the evolving state. As a next step, one shows $\partial_t \mathbf{p}_n + \partial_z \mathbf{e}_n = -\mathcal{F}_n^{\mathrm{dr}}$ through straightforward manipulations. First, one takes the time and space derivatives of both sides of Eqs. (S20) and (S21) respectively, and sums the two equations. The GHD equations in the space of root densities $\partial_t[(\partial_\lambda p_n)^{\mathrm{dr}}\vartheta_n(\lambda)] + \partial_x((\partial_\lambda E_n)^{\mathrm{dr}}\vartheta_n(\lambda)] + \partial_\lambda[\mathcal{F}^{\mathrm{dr}}\vartheta_n(\lambda)] = 0$ (notice that $\rho_n(\lambda) = \frac{1}{2\pi}(\partial_\lambda p_n)^{\mathrm{dr}}\vartheta_n(\lambda))$ are used for further simplifications. With this last step, one obtains $\partial_t \mathbf{p}_n + \partial_z \mathbf{e}_n = -\mathcal{F}_n(\lambda) + \sum_{n'} \int \mathrm{d}\lambda'\, \Phi_{n,n'}(\lambda-\lambda')\vartheta_{n'}(\lambda')[-\mathcal{F}_{n'}^{\mathrm{dr}}(\lambda')]$, and thus can identify $\partial_t \mathbf{p}_n + \partial_z \mathbf{e}_n = -\mathcal{F}_n^{\mathrm{dr}}$ as anticipated. Using now $\partial_z \mathbf{e}_n \to \partial_z \mathcal{H}_n(x, \mathbf{p})$, one can rewrite the GHD equations in an explicit symplectic form

$$\partial_t \bar{\vartheta}_{n,x}(\mathbf{p}) + \partial_{\mathbf{p}}\mathcal{H}_j(x, \mathbf{p})\partial_x \bar{\vartheta}_{n,x}(\mathbf{p}) - \partial_x \mathcal{H}_n(x, \mathbf{p})\partial_{\mathbf{p}}\bar{\vartheta}_{n,x}(\mathbf{p}) = 0\,. \quad \text{(S22)}$$

These are the Liouville's equations [15] in the phase space $(z, \mathbf{p})$ for a particle evolving with classical Hamiltonian $\mathcal{H}_n(z, \mathbf{p})$. The evolution can be seen in two ways: either as a function evolving in a time-independent phase space, or as if the coordinates are evolving with a fixed density background $\bar{\vartheta}_{n;t,z(t)}(\mathbf{p}(t)) = \bar{\vartheta}_{n;t=0,z}(\mathbf{p})$, where coordinates obey the equations of motion $\dot{z} = \partial_{\mathbf{p}}\mathcal{H}_n$ and $\dot{\mathbf{p}} = -\partial_z \mathcal{H}_n$. To prove Eq. (S19), the second interpretation is more convenient. Notice that the Liouville's theorem [15] guarantees that the phase-space volume $\mathrm{d}z\mathrm{d}\mathbf{p}$ is constant in time. We consider the left-hand side of Eq. (S19) and express it in the $(z, \mathbf{p})$ plane $\sum_n \int \mathrm{d}z\mathrm{d}\mathbf{p}\, \frac{p_n(\lambda_n(\mathbf{p}))}{2\pi}\partial_{\mathbf{p}}\mathcal{H}_n \bar{\vartheta}_n(\mathbf{p})$, where $\lambda_n(\mathbf{p})$ denotes the inverse change of variable $\lambda \to \mathbf{p}$. We use $\partial_{\mathbf{p}}\mathcal{H}_n = \dot{z}$ and straightforward identities reaching

$$\int \mathrm{d}z \sum_n \int \mathrm{d}\lambda\, p_n(\lambda)(\partial_\lambda E_n)^{\mathrm{dr}}\vartheta_n(\lambda) = \frac{\mathrm{d}}{\mathrm{d}t}\left(\sum_n \int \mathrm{d}z\mathrm{d}\mathbf{p}\, \frac{p_n(\lambda_n(\mathbf{p}))}{2\pi} z\bar{\vartheta}_n(\mathbf{p})\right) - \sum_n \int \frac{\mathrm{d}z\mathrm{d}\mathbf{p}}{2\pi}\frac{\mathrm{d}p_n(\lambda_n(\mathbf{p}))}{\mathrm{d}t} z\bar{\vartheta}_n(\mathbf{p})\,. \quad \text{(S23)}$$

We now use the chain rule to compute $\frac{\mathrm{d}p_n(\lambda_n(\mathbf{p}))}{\mathrm{d}t} = \partial_t[p_n(\lambda_n(\mathbf{p}))]\Big|_{\text{fixed }\mathbf{p}} + \dot{\mathbf{p}}\partial_{\mathbf{p}}(p_n\lambda_n(\mathbf{p}))$, then we use $\dot{\mathbf{p}} = -\partial_z\mathcal{H}_n$ and move to time derivatives at fixed λ by using $\partial_t[p_n(\lambda_n(\mathbf{p}))]\Big|_{\text{fixed }\mathbf{p}} = \partial_t p_n(\lambda_n(\mathbf{p})) - \partial_t\mathbf{p}_n\partial_{\mathbf{p}}[p_n(\lambda_n(\mathbf{p}))]$. Therefore, we can write $\frac{\mathrm{d}p_n(\lambda_n(\mathbf{p}))}{\mathrm{d}t} = \partial_t p_n(\lambda_n(\mathbf{p})) + \mathcal{F}_n^{\text{eff}}(\lambda_n(\mathbf{p}))\partial_{\mathbf{p}}(p_n(\lambda_n(\mathbf{p})))$. We notice that, in the Lieb Liniger model, $\partial_t p_n(\lambda) = 0$. Using this last identity in Eq. (S23) and rewriting it in terms of the rapidities, Eq. (S19) follows.

Supplementary References

[1] O. A. Castro-Alvaredo, B. Doyon, and T. Yoshimura, Emergent hydrodynamics in integrable quantum systems out of equilibrium, Physical Review X **6**, 041065 (2016).

[2] B. Bertini, M. Collura, J. De Nardis, and M. Fagotti, Transport in out-of-equilibrium XXZ chains: exact profiles of charges and currents, Physical Review Letters **117**, 207201 (2016).

[3] B. Doyon and T. Yoshimura, A note on generalized hydrodynamics: inhomogeneous fields and other concepts, SciPost Physics **2**, 014 (2017).

[4] A. Bastianello, V. Alba, and J.-S. Caux, Generalized hydrodynamics with space-time inhomogeneous interactions, Physical Review Letters **123**, 130602 (2019).

[5] R. Koch, A. Bastianello, and J.-S. Caux, Adiabatic formation of bound states in the one-dimensional Bose gas, Physical Review B **103**, 165121 (2021).

[6] M. Girardeau, Relationship between systems of impenetrable bosons and fermions in one dimension, Journal of Mathematical Physics **1**, 516 (1960).

[7] A. Lenard, One-dimensional impenetrable bosons in thermal equilibrium, Journal of Mathematical Physics **7**, 1268 (1966).

[8] A. Bastianello, M. Collura, and S. Sotiriadis, Quenches from bosonic Gaussian initial states to the Tonks-Girardeau limit: stationary states and effects of a confining potential, Physical Review B **95**, 174303 (2017).

[9] M. Takahashi, *Thermodynamics of one-dimensional solvable models* (Cambridge university press, Cambridge, 1999).

[10] R. Koch, J.-S. Caux, and A. Bastianello, Generalized hydrodynamics of the attractive non-linear Schrödinger equation, Journal of Physics A: Mathematical and Theoretical **55**, 134001 (2022).

[11] K. V. Kheruntsyan, D. M. Gangardt, P. D. Drummond, and G. V. Shlyapnikov, Pair correlations in a finite-temperature one-dimensional Bose gas, Physical Review Letters **91**, 040403 (2003).

[12] J.-S. Caux, B. Doyon, J. Dubail, R. Konik, and T. Yoshimura, Hydrodynamics of the interacting Bose gas in the quantum Newton cradle setup, SciPost Physics **6**, 070 (2019).

[13] Research data are freely available from Zenodo at doi.org/10.5281/zenodo.15426226.

[14] S. Tan, Generalized virial theorem and pressure relation for a strongly correlated Fermi gas, Annals of Physics **323**, 2987 (2008).

[15] V. I. Arnol'd, *Mathematical methods of classical mechanics*, Vol. 60 (Springer Science & Business Media, 2013).
