# Supplementary material for: Probing Bethe strings in an attractive one-dimensional Bose gas
Source: Nat Commun. 2026 Aug 29;17:10330. doi: 10.1038/s41467-026-76018-0 (PMC13623881; doi:10.1038/s41467-026-76018-0)
Supplement: Supplementary file 2 [file 41467_2026_76018_MOESM2_ESM.pdf]

nature portfolio

Peer Review File

Probing Bethe strings in an attractive one-dimensional Bose gas

Corresponding Author: Professor Hanns-Christoph Nagerl

This file contains all reviewer reports in order by version, followed by all author rebuttals in order by version.

Version 0:

Reviewer comments:

Reviewer #1

(Remarks to the Author)
Dear Editor,

The manuscript "Observing Bethe strings in an attractive Bose gas far from equilibrium" reports the observation of dense intermixtures of Bethe strings, multi-particle bound states predicted by the Bethe ansatz, in a one-dimensional attractive Bose gas. The authors employ ultracold cesium atoms confined in two-dimensional optical lattices and dynamically ramp the interaction from repulsive to attractive using a Feshbach resonance. This procedure drives the gas out of equilibrium and creates a mixture of bound states (Bethe strings) of various sizes.

Experimental signatures of Bethe strings are inferred from comparisons between 1D and 3D time-of-flight (TOF) expansions. In the attractive regime, the 3D release energy is found to be significantly larger than the 1D release energy, indicating conversion of binding energy upon release. Based on an uncertainty-principle argument, this difference suggests the formation of bound states containing up to six atoms. The experimental measurements show good agreement with theoretical predictions. The paper presents an exciting experimental route for probing few-body bound states in integrable 1D systems.

Major Comments
1. Indirect evidence and dependence on theory
The interpretation relies heavily on theoretical modeling. Although the title states "observing Bethe strings…" the experimental signatures, such as broadened momentum distributions, are linked to the presence of bound states primarily through theoretical arguments. Direct experimental evidence that a few atoms are bound and move together in the tubes is still missing. Independent confirmation could come from measurements of the bound-state size or binding energy, for instance through radiofrequency or Bragg spectroscopy, or from observations of collective bound motion. Author should reconsider the title.
The claim that the system supports intermixtures of many strings also requires clarification. Figure 3c shows the predicted distribution of string lengths, but the experimental data are fitted by a single string size with one binding energy. Are there direct evidences that multiple string sizes must coexist in the sample?

2. Connection to soliton physics
The formation of soliton chains in attractive 1D Bose gases has been well established. Although the authors cite the work by Randall Hulet's group, the conceptual connection is not discussed. Are Bethe strings related to quantized solitons, or can they aggregate into soliton-like structures? A brief comparison or discussion of this relationship would greatly clarify the physical picture.

3. Relation to few-body Efimov states
Previous experiments with caesium atoms from the same Innsbruck group have observed three- and four-body Efimov states in the attractive regime. The authors should comment on whether and how the Bethe strings studied here relate to those few-body bound states, given that both arise in the same atomic species and interaction domain.

4. Integrability breaking and experimental imperfections
The analysis assumes that the system is nearly integrable, yet experimental imperfections: tube inhomogeneity, transverse

excitations, and atom loss are non-negligible. The paper does not quantify how these effects might modify the dynamics or cause decay of bound states. The disagreement between experiment and GHD theory for strong attraction (Fig. 4) is attributed to such effects but not explicitly modeled. A more quantitative discussion of loss rates, string lifetimes, and tube-to-tube variation would help assess the robustness of the claimed Bethe-string ensemble.

5. Model dependence and fitting assumptions
The quantitative conclusions, such as the estimated typical string size, rely strongly on generalized hydrodynamics (GHD) modeling and on assumed initial conditions (temperature, atom number per tube). The fits between experiment and theory involve several adjustable parameters, particularly the initial 1D temperature and the crossover temperature (Supplementary Section 1). This flexibility may introduce model bias that masks discrepancies. Explicitly providing confidence intervals or robustness checks of the GHD fits with respect to parameter variations would improve transparency and credibility.

Minor Comments
A. For Bethe strings, the real part of the rapidity can in principle be both positive and negative. Figure 1a shows it is non-negative. Is that correct? Also, it is not accurate to describe \tilde{c} >0 as an “ideal gas.”

B. The parameter \tilde{c} is introduced in Fig. 1(a) but defined only later. Please define it when it first appears.

C. The term “rapidity” should be defined upon first mention, for example:
“Here \theta are the rapidities, i.e. quasi-momenta that parametrize the Bethe-ansatz eigenstates.”

D. The quantities \Delta z_n, a_\text{ho}, E_{1D}, and E_{3D} should also be defined when they first appear.

E. G_2 and the 1D Tan’s contact CCC should be explicitly defined in the main text.

Summary
This paper presents a significant and technically impressive advance in realizing attractive 1D Bose gases that support Bethe strings. However, the current evidence for multi-particle bound states remains indirect and theory-dependent. The manuscript would be substantially strengthened by a clearer connection to soliton and few-body physics, a more detailed discussion of integrability-breaking effects, and quantitative assessments of model assumptions. I will consider recommend publication after the authors satisfactorily address the above points.

Reviewer #2

(Remarks to the Author)
In the work “Observing Bethe strings in an attractive Bose gas far from equilibrium", the authors provide a first experimental realization of a stable intermixtures of many Bethe strings in a nearly integrable ultracold Bose gas. The intermixture of bosonic bound states is created by sweeping the interaction from repulsive to attractive, passing through the non-interacting point, in a non-equilibrium setting.\\The presence of Bethe strings, is proven using three independent methods.
Firstly, the condensate is loaded in a 3D crossed-beam optical dipole trap, resulting in an ensemble of 1D tubes with both longitudinal and transverse harmonic confinements. The presence of bound states is proven by measuring the release energy after respectively 1D and 3D expansion, and comparing the associated release energies. The results show that, while in the repulsive regime the two coincide, in the attractive regime the energy release during the 3D expansion exceeds the one measured for the 1D counterpart. In fact, it takes into account the binding energies of bound states, being preserved during the 1D evolution. The validity of the method is also enforced by the consistency with the theoretical predictions.\\The second method is based on the measure of momentum distribution after 1D expansion and with interaction strength ramped from the repulsive to the attractive regime. The experimentally measured dynamics is in agreement with the GHD time evolution. This experiment permits to examine how the temperature affects the formation of the bound states, by looking at the width of the quasiparticle's momentum distribution. The result is that, for increasing temperature, the kinetic energy of the system dominates over the interaction energy, reducing the formations of strings.\\Finally, they analise how the presence of Bethe strings modifies the
short-range correlations of the gas by looking at the Tan’s contact. The results, again, are in agreement with the theoretical predictions provided by GHD.\\In this work, a new state of quatum matter is observed at non-equilibrium, the observations and results are of high interest and quality. The observations, interpretations and conclusions are solid, being supported by the GHD theoretical predictions, even though the agreement doesn't look perfect in every regime. Also, the methodology and results are exposed in a clear way, accessible to a broad audience. I would like to recommend publication of the paper; however, I feel the writing of the paper could be improved if a few things are clarified further. Below I list these points.
\\begin{itemize}
\item In the paragraph: \textit{"We control the atom number in the BEC within the range of 2×10^4 to 6×10^4, with an uncertainty of 10%...This results in approximately 4000 isolated 1D tubes...We tune the average number of atoms per tube N between 7 and 30 by varying the density of the initial BEC."}
From a naive calculation, seems that the number of atoms per tube should be between 5 and 15 ($2-6\times 10^4/4000$). The authors should comment on or correct this point.

\item In the whole text the theoretical predictions are provided by Generalized Hydrodynamics, while the number of particles seems small ($7-30$). Why do you expect hydrodynamics to be predictive in this context?

\item In the first experimental prove, you observe the presence of Bethe strings by measuring the release energy for 1D and 3D expansion. You say: \textit{"The corresponding GHD simulations agree well with our measurements."}. I understand that

this is a non-equilibrium setting, since you ramped the interaction strength from the repulsive to attractive. From Supplementary Note 2, I also understand that by “GHD” you refer to Eq. (S1), which takes into account the weak integrability breaking induced by the trap and the effect of slow interaction changes.
However, this statement is somewhat confusing at first glance, since “GHD” typically refers to a fully integrable setting. I think it would be helpful to add a brief clarification in the main text, explicitly mentioning that the comparison is made with a GHD equation that includes weak integrability breaking due to the trap and slow interaction ramps.

\item In Fig.2 there is clearly a disagreement in the comparison with theory for $\tilde{c}\lesssim-1$ in the 3D expansion. Is this still due to finite-size effects, as in Fig.4?

\item An ending point is missing to the sentence \textit{"At the beginning of the ramp $f(p/\hbar)$ is bell-shaped with a full width at half maximum (FWHM) $\hbar\times 6.0(1)\mu m^{-1}$".}

\item About Fig.3b, could you comment why, at $35nK$, the experimental results seem completely insensitive to the presence of strings and the agreement with GHD becomes worse?
You state that \textit{"For increasing temperature, the kinetic energy of the system dominates over the interaction energy. This reduces the probability of forming larger bound states, and in turn reduces the average binding energy per particle."}. However, the observed behavior appears more dramatic than what GHD predicted.

\item In Fig.3c, what is it the reason why you decided to plot nP_n? I think that just P_n, being \textit{"the average number of strings of size n"}, would be a more intuitive observable. In that case, $P_n>1$ would indicate that a certain Bethe string is typically present.

\item I would appreciate some physical considerations about the results shown in Fig4. Why Tan's contact is increasing for increasing negative $\tilde{c}$? Such a discussion would give more significance to this final experimental observation.

\item Finally, mostly importantly it would be good to clarify in which quantum or semi-classical regime the experiments is. Would it be possible to reproduce the data also with a simulation of Nonlinear Schrödinger, Gross–Pitaevskii equation?
\end{itemize}

Reviewer #3

(Remarks to the Author)
I co-reviewed this manuscript with one of the reviewers who provided the listed reports. This is part of the Nature Communications initiative to facilitate training in peer review and to provide appropriate recognition for Early Career Researchers who co-review manuscripts.

Version 1:

Reviewer comments:

Reviewer #1

(Remarks to the Author)
Dear Editor,

The authors provide a professional and thoughtful response to my comments. They clarify several conceptual issues, including the relation to soliton physics and few-body states, add textual improvements to the manuscript, and partially address concerns regarding integrability breaking and model dependence. Their clarification of how the full string distribution enters the simulations is helpful, and the inclusion of limited confidence intervals improves transparency.

However, central concerns remain only partially resolved. In particular, the distinction between indirect evidence and a strong claim of “observation” is largely defended rather than reinforced by additional experimental validation. The argument for the coexistence of multiple string sizes continues to rely predominantly on modeling rather than on experimentally discriminating observables. The discussion of integrability breaking and fitting robustness, while expanded, remains largely qualitative, and the limited parameter-variation analysis at the level of 10% does not fully address concerns about model sensitivity.

While the experimental results are certainly compelling, I believe the work would benefit from a more critical stance toward the theoretical framework used to interpret the data. Much of the analysis is directed toward adapting the integrability-based model to explain the observations, rather than toward actively testing its validity and limits. Given the far-from-equilibrium and only approximately integrable regime explored, the manuscript would be significantly strengthened by explicitly probing limits of the theory and by identifying observables capable of discriminating between competing interpretations. For example, alternative frameworks such as few-body
N-body (N=3,4,5...) bound states, or modulation-instability–driven dynamics studied in related regimes (e.g., Hulet’s work and subsequent experiments such as 1703.07502 reporting agreement with NPSE) should be more critically examined as potential candidates.

Unambiguous signatures of Bethe strings, such as spectroscopic resolution of the predicted binding-energy spectrum scaling as n(n^2-1), or higher-order N-body correlations with the predicted structure, would substantially strengthen the claim. At present, the title “Observing Bethe strings in an attractive Bose gas…” appears stronger than what the experimental directness demonstrated. A formulation such as “Strong signatures of Bethe strings in a 1D attractive Bose gas” would more accurately reflect the current evidentiary basis.

Overall, the reply is careful and technically competent, but it only partially resolves the deeper concerns regarding experimental directness, model dependence, and the strength of the central claim. I recommend the publication of the paper with a title that better reflects the current evidence of the Bethe strings.

Reviewer #2

(Remarks to the Author)

The authors have addressed the majority of the previous concerns and significantly improved the clarity of the manuscript. Most of the requested revisions have been integrated effectively, and I believe only a few minor points now remain to be resolved before the paper is suitable for publication.

\begin{itemize}
\item \textit{"The dominant integrability breaking process in our experiment is three-body loss, which is not captured by GHD. Such loss is negligible in the repulsive phase, but in the attractive regime, as Bethe strings form and tighten upon increasing interactions three-body losses become appreciable."} This addition is of central importance, as it explains why the comparison between GHD and experimental data deviates in the attractive regime. However, given its significance, the authors should provide a more quantitative justification. Are three-body losses truly dominant compared to the effects of the longitudinal trap and transverse excitations? Or is just that they are not modeled within the GHD framework? Furthermore, in the repulsive regime, one would expect these losses to be subleading relative to other integrability-breaking effects; the authors should clarify this distinction.

\item Regarding the inset of Fig. 2, the average binding energy is compared with GHD and two exact string sizes. Could the authors clarify if this represents the binding energy per particle? While lines 234–250 present this as the total binding energy, the lower bound is defined as $\langle E_b\rangle \geq E_{b,n^*}/n^*$. The authors should ensure the terminology and scaling are consistent throughout the text.

\item Regarding my previous comment ($\#$6): the authors claim that less accurate modeling of the initial state at higher temperatures induces the discrepancies seen in Fig. 3b at 35 nK. I recommend addressing this explicitly in the main text (lines 298–310). Because there is a visible qualitative difference between the GHD predictions and experimental observations for this curve, the statement "Results from GHD are in good agreement with our experimental data" is currently insufficient and should be qualified.

\end{itemize}

Reviewer #3

(Remarks to the Author)
I co-reviewed this manuscript with one of the reviewers who provided the listed reports. This is part of the Nature Communications initiative to facilitate training in peer review and to provide appropriate recognition for Early Career Researchers who co-review manuscripts.

Version 2:

Reviewer comments:

Reviewer #1

(Remarks to the Author)
The authors have provided a detailed response and added valuable supplementary data regarding the 1D nature of the system. However, the fundamental disagreement regarding the strength of the central claim remains. The authors maintain that their experiment constitutes an "unquestionable observation" of Bethe strings. I remain not completely convinced that the measurement of an integrated quantity, the expansion energy, justifies this terminology. The main concerns are:

1. Spectral Specificity: A Bethe string is defined by a specific, quantized binding energy scaling as E_b ∝ n(n2−1). The expansion energy measured here is an average over a theoretical distribution of string sizes n. Without resolving the contributions of individual n-particle strings, or at least demonstrating the scaling Eb ∝ n^3, the measured "surplus energy" is at most consistent with the Bethe string manifold, but does not independently demonstrate discrete bound states or identify the manifold's internal structure.

2.The "Consistency" Trap: In many-body physics, "observation" typically implies the measurement of a unique signature that

excludes alternative descriptions. This is what I meant by “model sensitivity”. Here, the authors use Generalized Hydrodynamics (GHD) to fit the data. The agreement shows that the GHD framework (which supports Bethe strings) is a viable model for the data. It does not prove that the underlying entities are exclusively Bethe strings, rather than generic short-range bound clusters that would arise in any attractive 1D Bose gas, even in cases where integrability is significantly broken.

3. Stability as a proxy for identity: The authors claim that the stability during 1D expansion "directly probes this hallmark property (long lifetime) of the Bethe strings." While interesting, stability alone is also not a unique identifier. Many-body bound states in 1D are kinematically constrained; a "cluster" of atoms may persist simply due to the lack of available decay channels in a restricted geometry, e.g., solitons, regardless of whether it maps precisely onto a Bethe string solution of the Lieb–Liniger model.

4. Model Dependence: The authors state that they only fit two parameters (initial temperature and crossover temperature). However, the entire interpretation of the resulting energy curve relies on the assumption that the system precisely follows the string-charge distributions of GHD. If the experiment cannot independently verify the string distribution Pn in the manuscript, the claim of "observing" the strings becomes circular: the strings are built into the model to explain the energy, and the energy is then used to claim the observation of the strings.
The manuscript undoubtedly presents high-quality experimental work on attractive 1D gases. However, there is a formal scientific distinction between detecting a signature (greater momentum spread) consistent with a theory and observing the constituent quasi-particles (Bethe strings) of that theory.

Regarding the current title, to "Observe Bethe Strings" suggests that one can now directly detect quasi-particles with unique features that are exclusive to Bethe strings. Direct evidence should include either distinct spectral branches or, at minimum, n-body correlations. A good example is the “Experimental Observation of Bethe Strings” in Nature 554 (2018) 219, which achieves this standard.
I reiterate my recommendation: the paper is highly interesting and is suitable for publication if the claim is clarified. Suggested titles include: "Signatures of Bethe string dynamics in an attractive Bose gas far from equilibrium" or "Probing the energy of Bethe strings in a 1D attractive Bose gas."

Reviewer #2

(Remarks to the Author)
we thank the authors for their iteration of the manuscript, which is now ready for publication.

Reviewer #3

(Remarks to the Author)
I co-reviewed this manuscript with one of the reviewers who provided the listed reports. This is part of the Nature Communications initiative to facilitate training in peer review and to provide appropriate recognition for Early Career Researchers who co-review manuscripts.

Version 3:

Reviewer comments:

Reviewer #1

(Remarks to the Author)
I recommend publication of the paper with the revised title.

Open Access This Peer Review File is licensed under a Creative Commons Attribution 4.0 International License, which permits use, sharing, adaptation, distribution and reproduction in any medium or format, as long as you give appropriate credit to the original author(s) and the source, provide a link to the Creative Commons license, and indicate if changes were made.
In cases where reviewers are anonymous, credit should be given to 'Anonymous Referee' and the source.
The images or other third party material in this Peer Review File are included in the article's Creative Commons license, unless indicated otherwise in a credit line to the material. If material is not included in the article's Creative Commons license and your intended use is not permitted by statutory regulation or exceeds the permitted use, you will need to obtain permission directly from the copyright holder.
To view a copy of this license, visit https://creativecommons.org/licenses/by/4.0/

Response to the referees' comments on the manuscript:
Observing Bethe strings in an attractive Bose gas far from equilibrium

Dear Editor,

We thank you for considering our manuscript NCOMMS-25-68849-T *"Observing Bethe strings in an attractive Bose gas far from equilibrium"* and for sending us the referees' reports. These have allowed us to greatly improve the manuscript. In the following, we itemize the detailed response to the reviewers' comments (all reviewers' comments are in blue) and describe how we have updated the manuscript to meet their requests (changes are highlighted in purple color).

Response to reviewer 1:

The manuscript "Observing Bethe strings in an attractive Bose gas far from equilibrium" reports the observation of dense intermixtures of Bethe strings, multi-particle bound states predicted by the Bethe ansatz, in a one-dimensional attractive Bose gas. The authors employ ultracold cesium atoms confined in two-dimensional optical lattices and dynamically ramp the interaction from repulsive to attractive using a Feshbach resonance. This procedure drives the gas out of equilibrium and creates a mixture of bound states (Bethe strings) of various sizes.

Experimental signatures of Bethe strings are inferred from comparisons between 1D and 3D time-of-flight (TOF) expansions. In the attractive regime, the 3D release energy is found to be significantly larger than the 1D release energy, indicating conversion of binding energy upon release. Based on an uncertainty-principle argument, this difference suggests the formation of bound states containing up to six atoms. The experimental measurements show good agreement with theoretical predictions. The paper presents an exciting experimental route for probing few-body bound states in integrable 1D systems.

Our response: We thank referee 1 for his/her time in studying our manuscript in depth and for sharing the excitement for this new experimental venue. Before addressing the referee's various comments, we would like to point out that our claim of observing Bethe strings is based on a) binding energy measurements, see Fig. 2, b) correlation measurements, see Fig. 4, and c) comparison of the momentum distribution after 1D expansion with numerical data obtained via generalized hydrodynamics (GHD) simulations. We probe a system far from equilibrium and deeply in the quantum regime with strong interactions. Each of which is a theoretical challenge per-se, making the observed agreement remarkable.

Major Comments:

1. Indirect evidence and dependence on theory. The interpretation relies heavily on theoretical modeling. Although the title states "observing Bethe strings..." the experimental signatures, such as broadened momentum distributions, are linked to the presence of bound states primarily through theoretical arguments. Direct experimental evidence that a few atoms are bound and move together in the tubes is still missing. Independent confirmation could come from measurements of the bound-state size or binding energy, for instance through radio-frequency or Bragg spectroscopy, or from observations of collective bound motion. Author should reconsider the title. The claim that the system supports intermixtures of many strings also requires clarification. Figure 3c shows the predicted distribution of string lengths, but

the experimental data are fitted by a single string size with one binding energy. Are there direct evidences that multiple string sizes must coexist in the sample?

Our response: We think our measurements are in close correspondence to what the referee is asking for. Namely, the primary evidence we provide is indeed from the measurement of the (average) binding energy, which is a rather direct proof for the existence of cold-atom Bethe string states and their presence in our setup. Supporting evidence is given by the correlation measurement, i.e., of Tan's contact. Our theoretical modeling then strongly supports our claims. We agree with the referee that there are other interesting observables that one could probe, i.e., via RF or Bragg spectroscopy. This shall be addressed in future work.

All simulation data presented in the figures, including the inset of Fig. 2, have been computed considering the full distribution of string sizes, such as those presented in Fig. 3c. The inset of Fig. 2 additionally provides the energy computed with a single string size to give an estimate of bound-state sizes that are expected in our setup. The full momentum distribution after expansion (Fig. 3a) has a strong dependence on the whole string distribution.

In the resubmitted version of our manuscript, we have added a clarification at the end of the caption of Fig. 2. We have also added a more detailed explanation in lines 234-250.

In view of the arguments we present above, in our opinion, our experiment provides a first observation of Bethe strings in a cold-atom setup, we think the current title "Observing Bethe strings in an attractive Bose gas far from equilibrium" is an appropriate description of our results.

2. Connection to soliton physics. The formation of soliton chains in attractive 1D Bose gases has been well established. Although the authors cite the work by Randall Hulet's group, the conceptual connection is not discussed. Are Bethe strings related to quantized solitons, or can they aggregate into soliton-like structures? A brief comparison or discussion of this relationship would greatly clarify the physical picture.

   Our response: Within the validity of the classical limit (small interactions and large number of particles), which is very far from the parameters' regime of our experiment (see also point 9 raised by referee 2), the Lieb-Liniger model is well-approximated by the 1D Gross-Pitaevskii Equation (GPE) equation. Here Bethe strings of large size become the bright solitons of the 1D GPE. The semiclassical limit is achieved in the limit of small interactions $\tilde{c}$ and large string sizes, in such a way that the discrete spectrum of Bethe strings approaches

the continuum of classical solitons [1]. An important distinction of our setup compared with Professor Hulet's work on elongated atomic systems should be emphasized: besides the fact that we explore a far deeper quantum regime, in our experiment the stability of Bethe strings is due to the approximated integrability of our systems, whereas in Hulet's experiments soliton stability is guaranteed only below a certain size.

In the resubmitted version of our manuscript, we clarify the connection with soliton physics in line 3 of the abstract and lines 56-60, and 64-69 of the main text.

3. Relation to few-body Efimov states. Previous experiments with caesium atoms from the same Innsbruck group have observed three- and four-body Efimov states in the attractive regime. The authors should comment on whether and how the Bethe strings studied here relate to those few-body bound states, given that both arise in the same atomic species and interaction domain.

   Our response: The deepest bound Efimov trimer is expected to appear for values of the 3D scattering length around $-950a_0$ for Cs. According to the analysis in Reference [2], we expect such trimer to survive in our quasi-1D situation for our parameter range. In the current experiment, we never explore such a high scattering-length regime, so we do not expect the Bethe strings to display features of Efimov states in the range of parameters explored. Nevertheless, the reviewer's comment points to a very interesting question regarding the connection of the Bethe string trimer and the Efimov trimer that should be explored in further work, from the experimental and theoretical point of view.

   In the resubmitted version of the manuscript we have added further emphasis on Efimov states in the conclusion (see lines 383-386).

4. Integrability breaking and experimental imperfections. The analysis assumes that the system is nearly integrable, yet experimental imperfections: tube inhomogeneity, transverse excitations, and atom loss are non-negligible. The paper does not quantify how these effects might modify the dynamics or cause decay of bound states. The disagreement between experiment and GHD theory for strong attraction (Fig. 4) is attributed to such effects but not explicitly modeled. A more quantitative discussion of loss rates, string lifetimes, and tube-to-tube variation would help assess the robustness of the claimed Bethe-string ensemble.

   Our response: The referee correctly mentions the three main sources of integrability-breaking, which are now addressed in the manuscript.

In the revised version of the manuscript, we now include a clearer and more detailed discussion. See lines 217-233.

5. Model dependence and fitting assumptions. The quantitative conclusions, such as the estimated typical string size, rely strongly on generalized hydrodynamics (GHD) modeling and on assumed initial conditions (temperature, atom number per tube). The fits between experiment and theory involve several adjustable parameters, particularly the initial 1D temperature and the crossover temperature (Supplementary Note 1). This flexibility may introduce model bias that masks discrepancies. Explicitly providing confidence intervals or robustness checks of the GHD fits with respect to parameter variations would improve transparency and credibility.

   Our response:

   We agree with the referee that showing the sensitivity of numerical predictions upon varying the fitting parameters is helpful to the reader. However, GHD simulations in the attractive phase are very costly. Each simulation needs about two weeks on a powerful cluster. When searching for the best fit during this project, we focused on simulations in the repulsive side, which are quicker. Once we determined the best fit in the repulsive branch by tuning $T_{\rm 1D}$ and $T_{\rm cross\text{-}over}$, we continued the simulation only for the selected parameters in the attractive side.

   To meet the referee's request within a reasonable time scale, in the resubmitted version of our manuscript, we provide a confidence interval only for the data of Fig. 3a, obtained by varying the fitting parameters of 10%. We observe that the agreement between theory and experiment is still very good showing the robustness of our data. We also discuss this in the main text in lines 275-279.

Minor Comments:

1. For Bethe strings, the real part of the rapidity can in principle be both positive and negative. Figure 1a shows it is non-negative. Is that correct? Also, it is not accurate to describe $\tilde{c} > 0$ as an "ideal gas."

   Our response: The referee is right in pointing out that the real part of the Bethe strings' rapidities can have arbitrary values, also negative. The impression that they are positive

numbers is due to an unfortunate artistic choice. We have also changed the label of the ideal gas phase to avoid confusion.

We have now updated Fig. 1a to include also negative rapidity values. For the weakly interacting regime we have change the label from 'ideal gas' to 'quasi-condensate'.

2. The parameter $\tilde{c}$ is introduced in Fig. 1(a) but defined only later. Please define it when it first appears.

   Our response: The referee might have overlooked that the interaction parameter $\tilde{c}$ first appears in Eq. (1) and it is defined right below it. It then appears in Fig.1, which in the text is considered only after Eq. (1). So everything should be fine.

3. The term "rapidity" should be defined upon first mention, for example: "Here θ are the rapidities, i.e. quasi-momenta that parametrize the Bethe-ansatz eigenstates."

   Our response: Also here everything should be fine. The term rapidities is introduced right after Eq.(1) and subsequently used in Fig.1.

4. The quantities $\Delta z_n, a_{\text{ho}}$, E_{1D}, and E_{3D} should also be defined when they first appear.

   Our response: Similarly to the previous point, we carefully revised the manuscript once again and found that all the quantities $\Delta z_n, a_{\text{ho}}$, $\mathcal{E}_{\text{1D}}$, and $\mathcal{E}_{\text{3D}}$ have been properly defined the first time they appear.

5. G_2 and the 1D Tan's contact C should be explicitly defined in the main text.

   Our response: Tan's contact is defined from G_2 at line 273, but we overlooked to properly define G_2 and $g_2(0)$.

   In the resubmitted version, we now define these quantities in lines 313-314.

Summary: This paper presents a significant and technically impressive advance in realizing attractive 1D Bose gases that support Bethe strings. However, the current evidence for multi-particle bound states remains indirect and theory-dependent. The manuscript would be substantially strengthened by a clearer connection to soliton and few-body physics, a more detailed discussion of integrability-breaking effects, and quantitative assessments of model assumptions. I will consider recommend publication after the authors satisfactorily address the above points.

Our response: We are pleased that the referee deems our results “significant and technically impressive”. Following the referee’s suggestion, we have improved our manuscript to meet the suggestions and provide further details in this reply. We hope that with this revision the referee finds the manuscript suitable for publication in Nature Communications.

Response to reviewers 2 & 3:

In the work "Observing Bethe strings in an attractive Bose gas far from equilibrium", the authors provide a first experimental realization of a stable intermixtures of many Bethe strings in a nearly integrable ultracold Bose gas. The intermixture of bosonic bound states is created by sweeping the interaction from repulsive to attractive, passing through the non-interacting point, in a non-equilibrium setting.

The presence of Bethe strings, is proven using three independent methods. Firstly, the condensate is loaded in a 3D crossed-beam optical dipole trap, resulting in an ensemble of 1D tubes with both longitudinal and transverse harmonic confinements. The presence of bound states is proven by measuring the release energy after respectively 1D and 3D expansion, and comparing the associated release energies. The results show that, while in the repulsive regime the two coincide, in the attractive regime the energy release during the 3D expansion exceeds the one measured for the 1D counterpart. In fact, it takes into account the binding energies of bound states, being preserved during the 1D evolution. The validity of the method is also enforced by the consistency with the theoretical predictions.

The second method is based on the measure of momentum distribution after 1D expansion and with interaction strength ramped from the repulsive to the attractive regime. The experimentally measured dynamics is in agreement with the GHD time evolution. This experiment permits to examine how the temperature affects the formation of the bound states, by looking at the width of the quasiparticle's momentum distribution. The result is that, for increasing temperature, the kinetic energy of the system dominates over the interaction energy, reducing the formations of strings.

Finally, they analyze how the presence of Bethe strings modifies the short-range correlations of the gas by looking at Tan's contact. The results, again, are in agreement with the theoretical predictions provided by GHD.

In this work, a new state of quantum matter is observed at non-equilibrium, the observations and results are of high interest and quality. The observations, interpretations and conclusions are solid, being supported by the GHD theoretical predictions, even though the agreement doesn't look perfect in every regime. Also, the methodology and results are exposed in a clear way, accessible to a broad audience. I would like to recommend publication of the paper; however, I feel the writing of the paper could be improved if a few things are clarified further. Below I list these points.

Our response: We thank the referees 2 & 3 for praising our work and for deeming our results to

be of "high interest and quality", and "accessible to a broad audience". We are grateful that they support publication in Nature Communications after their minor concerns are properly accounted for. As before we now provide a detailed point-by-point answer, and report the changes in the manuscript that we have made to meet their request.

1. In the paragraph: *"We control the atom number in the BEC within the range of* 2×10^4 *to* 6×10^4*, with an uncertainty of* 10%*...This results in approximately* 4000 *isolated 1D tubes...We tune the average number of atoms per tube N between 7 and 30 by varying the density of the initial BEC."* From a naive calculation, seems that the number of atoms per tube should be between 5 and 15 ($2 - 6 \times 10^4/4000$). The authors should comment on or correct this point.

   Our response: The number of tubes are not fixed in the experiment to 4000. In fact, changing the density of the condensate sample inadvertently varies the number of populated tubes in the experiment. Evidently the atomic population in the 1D tubes is inhomogeneous. The number of atoms per tubes cannot be reproduced by taking the total number of atoms divided by the number of tubes. We detail our modeling procedure in Supplementary Note 1.

   In the revised manuscript, we now specify a range for the number of populated tubes (see lines 129, 139-140). For further clarification we have added a range for the estimate of the number of tubes in the main text. Additionally we included the formula that we use to calculate N in Supplementary Note 1.

2. In the whole text the theoretical predictions are provided by Generalized Hydrodynamics, while the number of particles seems small ($7 - 30$). Why do you expect hydrodynamics to be predictive in this context?

   Our response: Indeed, it is remarkable that GHD is already predictive with such a low number of particles. From the theoretical viewpoint, the lower bounds on the particle's density for GHD to be valid is still unknown. However, this question has been experimentally investigated in the repulsive regime [3], showing that a number of atoms as small as $\simeq 11$ in each tubes already gave good agreement with GHD. In this reference, it is argued that while for such a low number of particles fluctuations would be important, GHD still captures the average dynamics, which is the only measurable quantity due to the averaging across several tubes. We expect such a mechanism to be at work in our setup as well, even though

the presence of Bethe strings arguably needs for more particles than the repulsive regime, since GHD works in the limit when each Bethe string is thermodynamically populated. Investigating the validity regime of GHD in the presence of Bethe strings is an intriguing future research direction.

In the revised manuscript, we now comment on the validity of GHD with a low number of particles in lines 217-233.

3. In the first experimental prove, you observe the presence of Bethe strings by measuring the release energy for 1D and 3D expansion. You say: *"The corresponding GHD simulations agree well with our measurements."*. I understand that this is a non-equilibrium setting, since you ramped the interaction strength from the repulsive to attractive. From Supplementary Note 2, I also understand that by "GHD" you refer to Eq. (S1), which takes into account the weak integrability breaking induced by the trap and the effect of slow interaction changes. However, this statement is somewhat confusing at first glance, since "GHD" typically refers to a fully integrable setting. I think it would be helpful to add a brief clarification in the main text, explicitly mentioning that the comparison is made with a GHD equation that includes weak integrability breaking due to the trap and slow interaction ramps.

   Our response: While it is true that GHD started as a hydrodynamic description to describe inhomogeneous states evolved with an exactly integrable Hamiltonian, the field developed much since the first seminal papers to include diverse sources of integrability-breaking, in particular traps (see eg. Reference [4] for a recent review). However, we agree with the referees that it is important for the broad readership to explicitly mention that GHD accounts for the inhomogeneous trap and time-dependent interactions: this is also connected with Point 4 raised by referee 1.

   In the revised version of the manuscript, we comment on this point at lines 217-233.

4. In Fig. 2 there is clearly a disagreement in the comparison with theory for $\tilde{c} \lesssim -1$ in the 3D expansion. Is this still due to finite-size effects, as in Fig. 4?

   Our response: referees 2 & 3 are correct. In the attractive phase, large strings give the largest contribution to the energy, but they are also most likely to be affected by finite-size effects and integrability-breaking corrections. Finite-size effects are due to the fact that, when considering large strings, a larger number of atoms is needed to reach the thermodynamic limit. We can make an oversimplified example: if one considers 50 atoms all bounded in

2-strings, one would have 25 fundamental excitations in the system. If, instead, one considers two strings of 25 particles, then one would have only 2 fundamental excitations in the system, arguably very far from the thermodynamic limit. Also, as mentioned in the main text, GHD predicts a substantial contribution from Bethe strings larger than the average number of particles in the tubes.

Concerning integrability-breaking terms, also in connection with point 4 raised by referee 1, large strings are more likely to be affected by integrability-breaking terms through three-body losses. The three-body loss increases with stronger attractive interaction, resulting in more atom loss and in lowering of the average binding energy as observed in the experiment with respect to theoretical predictions. This trend is qualitatively observed in our comparisons.

In the revised version of the manuscript, we comment on the finite-size effects and integrability breaking at lines 217-233.

5. An ending point is missing to the sentence *"At the beginning of the ramp $f(p/\hbar)$ is bell-shaped with a full width at half maximum (FWHM) $\hbar \times 6.0(1)$ μm^{-1}"*.

   Our response: In the revised version of the manuscript, we added a full stop to the end of the sentence *"At the beginning of the ramp $f(p/\hbar)$ is bell-shaped with a full width at half maximum (FWHM) $\hbar \times 6.0(1)$ μm^{-1}"*.

6. About Fig. 3b, could you comment why, at 35 nK, the experimental results seem completely insensitive to the presence of strings and the agreement with GHD becomes worse? You state that *"For increasing temperature, the kinetic energy of the system dominates over the interaction energy. This reduces the probability of forming larger bound states, and in turn reduces the average binding energy per particle."*. However, the observed behavior appears more dramatic than what GHD predicted.

   Our response: GHD provides a good quantitative agreement with all experimental data, but we agree that the agreement shown in the hotter dataset of Fig. 3b (35 nK) is worse. We believe this is mainly due to our modeling of the initial state. As explained in Supplementary Note 1, we assume at the beginning of the protocol all the tubes are at thermal equilibrium with a unique T_{1D} temperature, their population being determined by a unique parameter that we call $T_{\text{cross-over}}$. This approximation is currently the best modeling we can provide, but in practice one could expect deviations from it, both in the tube population and even in the 1D temperature of each of them. The very fundamental approximation of consider-

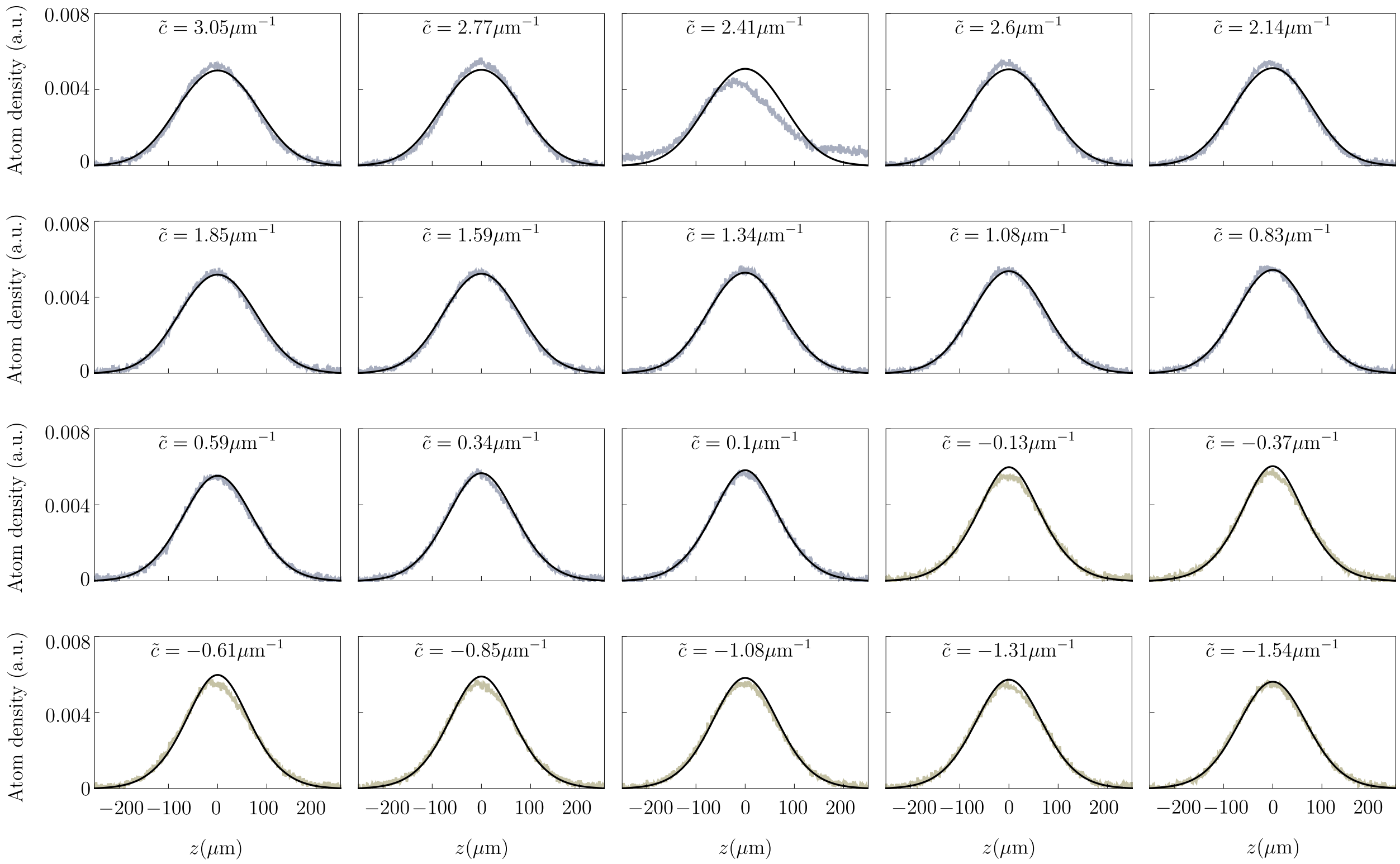

FIG. 1. **Momentum distribution after 1D expansion for the 35 nK dataset.** Distributions are shown for various values of the interaction parameter $\tilde{c}$ at the end of the ramp as indicated, from the repulsive (left, blue) to the attractive (right, yellow) regime. Each distribution is the mean of five repetitions and the shaded region corresponds to the standard error. The GHD simulations are given by the solid curves.

ing a thermal Gibbs ensemble for the initial 1D tubes could have corrections, since in 1D thermalization is suppressed. In practice, we see that this modeling provides an accurate description of the coldest dataset, but it shows corrections at hotter temperatures.

In Fig. 1, we provide an example of the comparison of the momenta distribution similarly for Fig. 3a, but here focusing on the 35-nK dataset (to be compared with Fig. S7 in the supplementary information to our manuscript).

In the revised version of our manuscript, we strengthen the discussion on the assumptions in the modeling of the initial states better highlighting possible source of errors. Furthermore, we updated Fig. S7 to include also the dataset shown above, to give the reader a better insight on the quality of the agreement of experimental data and GHD in the whole parameters' range.

7. In Fig. 3c, what is it the reason why you decided to plot nP_n? I think that just P_n, being *"the average number of strings of size n"*, would be a more intuitive observable. In that case, $P_n > 1$ would indicate that a certain Bethe string is typically present.

   Our response: We chose to show the participation nP_n since, while P_n is the typical probability of populating a string of size n, it contains n particles and so, generally speaking, it has n times the contribution of a $1-$string for those observables that are proportional to the number of atoms. For example, our data are renormalized to the average atom number per tube, hence $\sum_n nP_n = N$. Furthermore, plotting P_n would artificially show small tails, giving the wrong impression that large strings do not contribute much, and overall make the plot less readable.

8. I would appreciate some physical considerations about the results shown in Fig 4. Why Tan's contact is increasing for increasing negative $\tilde{c}$? Such a discussion would give more significance to this final experimental observation.

   Our response: The average Tan's contact is defined as $\tilde{c}^2 G_2$. The prefactor $\tilde{c}^2$ already helps the curve growing departing from $\tilde{c} = 0$ in both directions. Furthermore, G_2 behaves as follows: for $\tilde{c} = 0$, G_2 reaches a constant. For $\tilde{c} \to \infty$, G_2 goes to zero in such a way Tan's contact goes to a constant [5]. For $\tilde{c} < 0$, bound states are formed and they becomes tighter for stronger interactions, increasing the correlated density G_2. So, $\tilde{c}^2 G_2$ becomes very large for attractive interactions.

   We now clarify this point at lines 345-348 of the resubmitted manuscript.

9. Finally, mostly importantly it would be good to clarify in which quantum or semi-classical regime the experiments is. Would it be possible to reproduce the data also with a simulation of Nonlinear Schrödinger, Gross–Pitaevskii equation?

   Our response: We are exploring a deeply quantum regime where classical simulations with the Gross-Pitaevskii (GPE) (also referred to as Non-Linear Schrödinger equation (NLSE)) are not valid. The semiclassical limit is achieved for large temperature, large number of particles and small interactions: this is quantified by the parameters $\gamma = mg/(\hbar^2 d)$ and $\Theta = 2\hbar^2 k_{\mathrm{B}} T_{1\mathrm{D}}/(mg^2)$ (see eg. Fig. 2b in [6], where in our units the conventional interaction g is $g = \hbar^2 \tilde{c}/m$). For the parameters of Fig. 3a, for example, we can estimate these quantities on a representative tube of 15 atom discussed in Fig. S6. Using as an order of magnitude $\tilde{c} = 1\ \mu\mathrm{m}^{-1}$ and the density in the center of the trap $n = 1\ \mu\mathrm{m}^{-1}$, we obtain $\gamma \simeq 1$ and

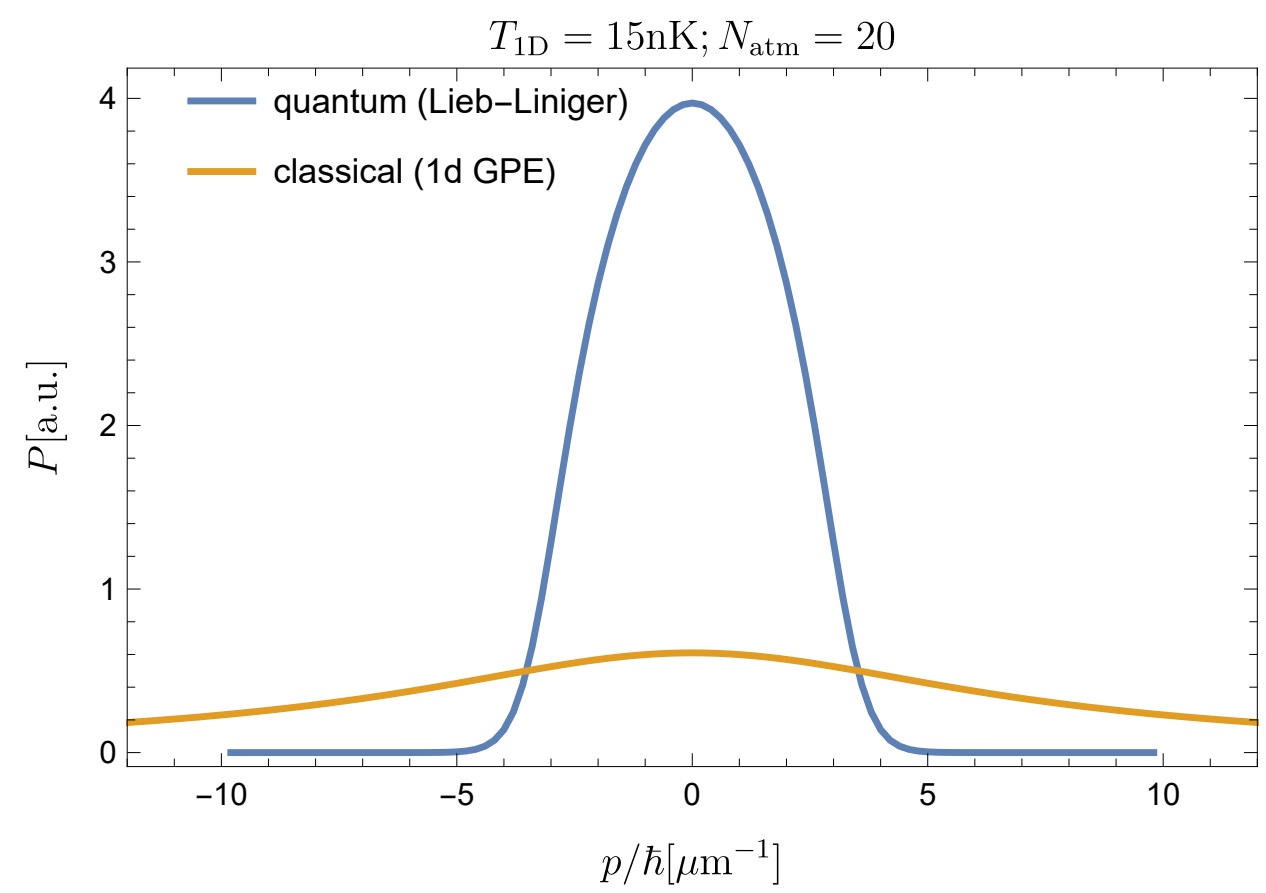

FIG. 2. **Theoretical momentum distribution after 1D expansion.** Distributions are obtained using Lieb-Liniger (blue) and NLSE (yellow) for a single tube of 15 particles.

$\Theta \simeq 8$, which are within the strongly interacting regime in Fig. 2b in [6].

To further strengthen this point, in Fig. 2 of this reply, we plot the theoretical momentum distribution after 1D expansion obtained for a tube of 15 particles in the quantum regime (using the Lieb-Liniger) and the same quantities computed, with the same parameters, in the NLSE (see Reference [7] for the equations governing the thermodynamics). We here focus on the initial state only, i.e., at the beginning of the ramp in the repulsive phase. As expected, the two curves are strikingly different, showing we are exploring the deep quantum regime.

Notice that in the attractive phase, the convergence of quantum Lieb-Liniger to the classical NLSE is even slower, as the discrete spectrum of the Bethe strings must reproduce the continuous energy spectrum of the classical solitons, see Reference [1] for a detailed discussion.

In the revised version of our manuscript, we now emphasize that our experiment probes the deep quantum regime at lines 146-150.

---

[1] R. Koch, J.-S. Caux, and A. Bastianello, Generalized hydrodynamics of the attractive non-linear Schrödinger equation, Journal of Physics A: Mathematical and Theoretical **55**, 134001 (2022).

[2] J. Levinsen, P. Massignan, and M. M. Parish, Efimov trimers under strong confinement, Phys. Rev. X **4**, 031020 (2014).

[3] N. Malvania, Y. Zhang, Y. Le, J. Dubail, M. Rigol, and D. S. Weiss, Generalized hydrodynamics in strongly interacting 1D Bose gases, Science **373**, 1129 (2021).

[4] B. Doyon, S. Gopalakrishnan, F. Møller, J. Schmiedmayer, and R. Vasseur, Generalized hydrodynamics: A perspective, Phys. Rev. X **15**, 010501 (2025).

[5] D. M. Gangardt and G. V. Shlyapnikov, Stability and phase coherence of trapped 1D Bose gases, Phys. Rev. Lett. **90**, 010401 (2003).

[6] M. Schemmer, I. Bouchoule, B. Doyon, and J. Dubail, Generalized hydrodynamics on an atom chip, Phys. Rev. Lett. **122**, 090601 (2019).

[7] G. D. V. D. Vecchio, A. Bastianello, A. D. Luca, and G. Mussardo, Exact out-of-equilibrium steady states in the semiclassical limit of the interacting Bose gas, SciPost Phys. **9**, 002 (2020).

Response to the referees' comments on the manuscript:
Observing Bethe strings in an attractive Bose gas far from equilibrium

Dear Editor,

We thank you for considering our manuscript NCOMMS-25-68849-T *"Observing Bethe strings in an attractive Bose gas far from equilibrium"* and for sending us the referees' reports. In the following, we itemize the detailed response to the reviewers' comments, giving all of the referee's comments in blue and our responses in black.

Response to reviewer 1:

The authors provide a professional and thoughtful response to my comments. They clarify several conceptual issues, including the relation to soliton physics and few-body states, add textual improvements to the manuscript, and partially address concerns regarding integrability breaking and model dependence. Their clarification of how the full string distribution enters the simulations is helpful, and the inclusion of limited confidence intervals improves transparency.

We are grateful to the reviewer for acknowledging the improvements we made in response to their report. We believe his/her constructive criticism helped us in communicating our results even more clearly than before.

However, central concerns remain only partially resolved. In particular, the distinction between indirect evidence and a strong claim of "observation" is largely defended rather than reinforced by additional experimental validation. The argument for the coexistence of multiple string sizes continues to rely predominantly on modeling rather than on experimentally discriminating observables.

We respectfully disagree with the reviewer on this point, and we believe that Bethe strings are unquestionably observed in our experiment, even though not individually resolved. In particular, Fig. 2 shows without any need for theoretical support the presence of bound states in the attractive regime, which are absent for repulsive interactions, and quantitatively measure their average binding energy. Theory is used to further support the correctness of this interpretation in a quantitative manner, but experimental data stands alone in showing the formation of bound states. We concede that a resolution of the individual Bethe strings would have further characterized the system, but it would have not changed the core message of our results, neither added critical information on the claim of "observing Bethe strings". Further in the report, the reviewer gives suggestions to better characterize the gas of Bethe strings. We gratefully acknowledge their input and provide a careful answer.

The discussion of integrability breaking and fitting robustness, while expanded, remains largely qualitative, and the limited parameter-variation analysis at the level of 10% does not fully address concerns about model sensitivity. While the experimental results are certainly compelling, I believe the work would benefit from a more critical stance toward the theoretical framework used to interpret the data. Much of the analysis is directed toward adapting the integrability-based model to explain the observations, rather than toward actively testing its validity and limits.

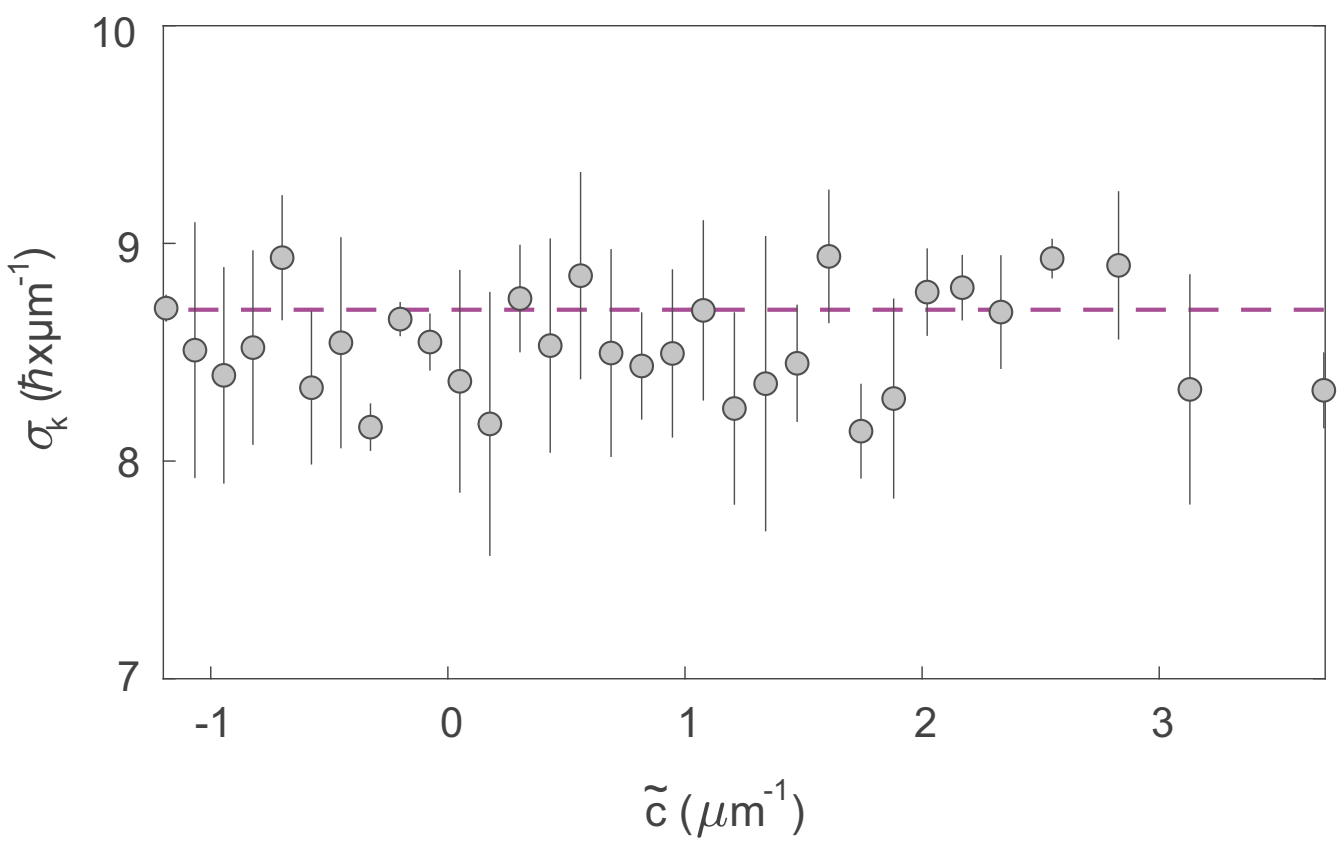

FIG. 1. Absence of transverse excitations. Typical transverse Gaussian width of the cloud σ_x as a function of interaction strength for $t_{1D} = 8$ ms, and $t_{3D} = 22.1$ ms. The transverse width of the sample remains within error of the expected ground-state momentum width (dashed line) for all interaction strengths, thus indicating that no transverse modes are populated throughout this protocol.

Without further elaboration, it is difficult for us to precisely assess what the reviewer means with "model sensitivity", but nonetheless we try to provide a complete answer on the variables in our modeling.

1. The microscopic theoretical model is the Lieb-Liniger model with a trapping potential. The "free parameters" in this model are the interaction strength and the trap frequency. Both of them can be determined experimentally. The trap frequency is experimentally measured with high accuracy. Furthermore, we explicitly checked anharmonic corrections computed from the knowledge of the size of the laser beam, and they did not appreciably alter the result. The interaction strength is also experimentally well-characterized through Feshbach resonances as discussed in the supplementary material.

2. We have experimentally verified that the approximation of having a one-dimensional system is reliable. We find that throughout the whole interaction ramp the transverse width of the cloud remains constant within experimental error (see Fig. 1). Moreover, it agrees with the expected theoretical value of a gas in the ground state. In the revised version of our manuscript, we now provide this measurement in the supplementary material as a benchmark for the one-dimensional approximation.

3. We remark that the GHD framework is explicitly meant to describe far-from-equilibrium states as the one engineered in our experiment, and it explicitly incorporates *i)* the effect of

the trapping potential, *ii)* the effect of interaction changes within the repulsive and attractive regimes, and *iii)* the excitations of Bethe strings when crossing the non-interacting point. All these effects are fixed by analytic formulas in GHD and not through fitting parameters. This modeling of trapping potentials has been previously validated both in theory through simulations and in experiments (see refs in the manuscript), while the effect of interaction strength changes and Bethe string bound states has been theoretically validated [JPhysA 55 (13), 134001 2022]. In this respect, our experiment also provides the first experimental validation of GHD with time-dependent interaction strength and formation of bound states. The only fitting parameters in our modeling are the one-dimensional temperature of the *initial* state, and the crossover temperature that affects the atom population across the tubes. We stress that the compelling agreement in Fig. 3a is obtained with only two fitting parameters, of which we show the 10% parameter variation.

4. The predominant effect not included in GHD is atom loss. We agree with the reviewer that including this effect would ideally improve the comparison. However, to our knowledge no theoretical framework for GHD with atom losses exists in the attractive regime, with the only related work being a single theoretical paper in the repulsive regime [SciPost Phys. 9, 044 (2020)], where losses are negligible in our setup. A quantitative treatment is therefore not currently possible, and we can only address losses at a qualitative level. We have added an approximate model of the loss process to Supplementary Note 4. The measured atom loss agrees with our modeling with a single fitting parameter. The fitted value is in good agreement known loss rates for Cs at the relevant magnetic field, further supporting our interpretation.

5. In view of the points above, we do not understand the review's criticism "Much of the analysis is directed toward adapting the integrability-based model to explain the observations". Indeed, we "adapted the model" just by fitting two parameters (1D and crossover temperatures), and from them everything is determined in our theoretical model.

Given the far-from-equilibrium and only approximately integrable regime explored, the manuscript would be significantly strengthened by explicitly probing limits of the theory and by identifying observables capable of discriminating between competing interpretations. For example, alternative frameworks such as few-body N-body ($N = 3, 4, 5...$) bound states, or modulation-instability–driven dynamics studied in related regimes (e.g., Hulet's work and subsequent experiments such as

1703.07502 reporting agreement with NPSE) should be more critically examined as potential candidates.

We stress that GHD has been devised with the purpose of describing far-from-equilibrium systems, where integrability is weakly broken. The quantitative comparison with experimental data is remarkable. We agree that it would have been interesting to compare predictions from GHD with alternative (and qualitatively different) theoretical interpretations. However, to the best of our knowledge, there are no other such interpretations. Indeed, elaborating on the reviewer inputs:

1. "...alternative frameworks such as few-body N-body ($N = 3, 4, 5...$) bound states...". This does not seem an alternative interpretation to us, since Bethe strings are indeed bound-states. Aside from one-dimensional physics described by Lieb-Liniger, if three-dimensional bound states were to be formed they would be lost from the trap, and their binding energy not visible (e.g. in Fig. 2). Hence, we do not see any other interpretation than having excited the bound states of the Lieb-Liniger model, i.e., the Bethe strings. Maybe, the reviewer suggests to investigate what is the effect of changing the size of the excited Bethe strings (or bound states) in the experimental observation. This is indeed what Fig. 3b aims to by tuning the initial temperature. Hotter (and so faster) particles are less likely to form bound states, diminishing their impact on the momentum distribution after 1D expansion. Our observations are quantitatively reproduced by theory.

2. "... or modulation-instability–driven dynamics studied in related regimes (e.g., Hulet's work and subsequent experiments such as 1703.07502 reporting agreement with NPSE)...". We thank the reviewer for pointing out this analogy that indeed we had overlooked and that connects to a comment in the previous report by the same reviewer. In the revised version of our manuscript, we highlight the important connection between our work and modulation instability in NLSE at lines 60-63, and 68-69. Modulation instability is the formation of solitons in the attractive NLSE (of which NPSE is a more accurate, but non-integrable, extension applicable to cold atoms) starting from homogeneous conditions seeded with small noise. Since the quantum Lieb-Liniger model and the classical NLSE are connected through the semiclassical limit, and Bethe strings become bright solitons in that limit. The modulation instability problem corresponds to the classical limit of a "quantum quench" where, in the ideal case, the ground state of the homogeneous non-interacting Bose gas is suddenly evolved with the attractive Hamiltonian. This problem has been studied in [Phys. Rev. Lett. 116, 070408 (2016)] and it is cited in our reference list. However, it is not directly

applicable to our experimental setup due to the inhomogeneity of the trapping potential and finite-temperature fluctuations. If instead of suddenly quenching interactions one uses a slow protocol, GHD can be used [PRB 103 (16), 165121 2021] on a much wider parameter set that includes our experiment. Hence, in summary, the interpretation offered in our work is not in contradiction with modulation instability, but it is just the quantum version of that providing one replaces "NLSE"→"Lieb-Liniger" and gently changes interactions rather than considering an abrupt evolution. We stress that changing the interaction ramp would arguably change the population of Bethe strings, but without altering the qualitative physics. For the sake of the reviewer's curiosity, slow interactions strength changes from repulsive to attractive have been studied within NLSE in the following reference: [JPhysA 55 (13), 134001 2022].

It is worth noticing that as the NPSE is non-integrable, the dynamics will spontaneously tend to form larger solitons with increasing binding energy also without changing interactions. Our observations are incompatible with this effect, as experimental data are well-reproduced by theory keeping the population of Bethe strings fixed, as predicted by GHD. However, our experiment works in a deeply quantum regime, far beyond the applicability of mean field semiclassical approaches such as the NLSE and its more refined versions (NPSE).

In summary, we do not think the interpretations suggested by the reviewer are alternative to our modeling, but on the contrary offer the same understanding expressed in a different language.

Unambiguous signatures of Bethe strings, such as spectroscopic resolution of the predicted binding-energy spectrum scaling as $n(n^2 - 1)$, or higher-order N-body correlations with the predicted structure, would substantially strengthen the claim. At present, the title "Observing Bethe strings in an attractive Bose gas. . . " appears stronger than what the experimental directness demonstrated. A formulation such as "Strong signatures of Bethe strings in a 1D attractive Bose gas" would more accurately reflect the current evidentiary basis. Overall, the reply is careful and technically competent, but it only partially resolves the deeper concerns regarding experimental directness, model dependence, and the strength of the central claim. I recommend the publication of the paper with a title that better reflects the current evidence of the Bethe strings.

We see this point somewhat differently from the reviewer, who suggests that our observation constitutes only ambiguous evidence of Bethe strings. The referee is correct in noting that the release energy measurement does not probe the energy of strings of individual sizes; however, much

like RF, or modulation spectroscopy probe the energy of strings of a single size, the release energy measurement probes the collective energy of the strings. Furthermore, an important characteristic of Bethe strings that distinguishes them from other bound states is their long lifetime. Neither RF nor modulation spectroscopy are sensitive to the lifetime of the measured states. In contrast, the expansion measurement necessarily requires the bound states to remain stable throughout the 1D expansion, and thereby directly probes this hallmark property of the Bethe strings. We therefore believe that our measurement constitutes unambiguous experimental evidence for the presence of Bethe strings, and that the current title is appropriate.

We thank the reviewer once again for his/her thorough report and detailed comments that spur us to further improve our manuscript. We hope the reviewer can now endorse the publication of our work in Nature Communications.

Response to reviewers 2 & 3:

The authors have addressed the majority of the previous concerns and significantly improved the clarity of the manuscript. Most of the requested revisions have been integrated effectively, and I believe only a few minor points now remain to be resolved before the paper is suitable for publication.

We thank the reviewers 2 & 3 for their suggestions and critical feedback. We are delighted the reviewers 2 & 3 are satisfied by the improvements in our manuscript and by its enhanced clarity. We thank the reviewers for deeming our manuscript suitable for publication. Below, we address the remaining minor points.

- *"The dominant integrability breaking process in our experiment is three-body loss, which is not captured by GHD. Such loss is negligible in the repulsive phase, but in the attractive regime, as Bethe strings form and tighten upon increasing interactions three-body losses become appreciable."* This addition is of central importance, as it explains why the comparison between GHD and experimental data deviates in the attractive regime. However, given its significance, the authors should provide a more quantitative justification. Are three-body losses truly dominant compared to the effects of the longitudinal trap and transverse excitations? Or is just that they are not modeled within the GHD framework? Furthermore, in the repulsive regime, one would expect these losses to be subleading relative to other integrability-breaking effects; the authors should clarify this distinction.

  We thank the reviewers for this comment, which helps us better clarifying further details of our theoretical modeling. He/she mentions three diverse sources of integrability breaking: a) losses, b) longitudinal trap, c) transverse excitations. Let us first address point a), i.e., losses. We experimentally observe that, in the repulsive phase, losses are small. We therefore expect that many-body losses coming from the scattering of unbounded particles are small. In the attractive regime, tight bound states are formed and these are expected to enhance many-body losses within the following picture. As the bound state binding energy grows, the bound state becomes tighter and many-body losses get enhanced. While this picture, which was already present in the previous version of the manuscript, is intuitively sounding, its theoretical modeling is an open problem for the scientific community. We nevertheless make an attempt at modeling the loss process, now added to Supplementary Note 4. We are able to recover the observed particle loss with a single fitting parameter with the meaning of a three-body loss coefficient. The fitted value is in good agreement with known loss rates

for Cs at the relevant magnetic field.

Concerning point b), i.e., the longitudinal trap, its effect is explicitly included in the GHD framework through the corresponding force terms. In our analysis we employ Euler-scale GHD, which is appropriate for the experimental regime considered. While the total evolution time (up to 130 ms) exceeds a single trap period (∼35 ms), it corresponds to only ∼3–4 oscillation periods. In this regime, Euler-scale GHD is expected to remain quantitatively accurate, whereas diffusive corrections are known to become relevant only at substantially longer timescales. We therefore do not expect diffusion to play a significant role in our experiment.

Finally for point c), i.e., transverse excitations, we have experimentally verified that transverse modes are not excited during the protocol by measuring the transverse momentum width, which remains approximately constant. In Fig. 1 we show a typical dataset of the transverse Gaussian width as a function of interaction strength. For further clarification of the text, we have added this figure to Supplementary Note 3.

Since *i)* the former manuscript already stated that GHD captures the effect of the longitudinal potential and *ii)* already discussed the role of three-body losses, and why they are enhanced in the attractive regime, in the resubmitted version we provide extra comments on the validity of the 1D regime at lines 165-167, and show the ToF measurements in the transverse direction in the supplementary material.

- Regarding the inset of Fig. 2, the average binding energy is compared with GHD and two exact string sizes. Could the authors clarify if this represents the binding energy per particle? While lines 234–250 present this as the total binding energy, the lower bound is defined as $\langle E_b \rangle \geq E_{b,n^*}/n^*$. The authors should ensure the terminology and scaling are consistent throughout the text.

  We thank the reviewer for pointing out the lack of clarity in our caption. In the manuscript, we consistently renormalize the momentum distribution to unity. Therefore, the measured average energy is, indeed, the average energy per particle. To avoid confusion, in the caption of Fig. 2 we corrected the first sentence from "Release energy $\mathcal{E}_{1D}$..." to "Release energy per particle $\mathcal{E}_{1D}$...".

- Regarding my previous comment (#6): the authors claim that less accurate modeling of the initial state at higher temperatures induces the discrepancies seen in Fig. 3b at 35 nK. I

recommend addressing this explicitly in the main text (lines 298–310). Because there is a visible qualitative difference between the GHD predictions and experimental observations for this curve, the statement "Results from GHD are in good agreement with our experimental data" is currently insufficient and should be qualified.

We welcome the reviewers' suggestion and add explicitly such a comment at lines 298-300.

We thank reviewers 2 & 3 for their time, careful assessment of our work, and their critical suggestions in improving our manuscript. We gladly address their comments and we hope they can now support the publications of our results in Nature Communications.

Response to the referees' comments on the manuscript:
Observing Bethe strings in an attractive Bose gas far from equilibrium

Dear Editor,

We thank you for considering our manuscript NCOMMS-25-68849-T *"Observing Bethe strings in an attractive Bose gas far from equilibrium"* and for sending us the referees' reports. In the following, we itemize the detailed response to the reviewers' comments, giving all of the referee's comments in blue and our responses in black.

Response to reviewer 1:

The authors have provided a detailed response and added valuable supplementary data regarding the 1D nature of the system. However, the fundamental disagreement regarding the strength of the central claim remains. The authors maintain that their experiment constitutes an "unquestionable observation" of Bethe strings. I remain not completely convinced that the measurement of an integrated quantity, the expansion energy, justifies this terminology. The main concerns are:

1. Spectral Specificity: A Bethe string is defined by a specific, quantized binding energy scaling as $E_{\mathrm{b}} \propto n(n^2-1)$. The expansion energy measured here is an average over a theoretical distribution of string sizes n. Without resolving the contributions of individual n-particle strings, or at least demonstrating the scaling $E_{\mathrm{b}} \propto n^3$, the measured "surplus energy" is at most consistent with the Bethe string manifold, but does not independently demonstrate discrete bound states or identify the manifold's internal structure.

2. The "Consistency" Trap: In many-body physics, "observation" typically implies the measurement of a unique signature that excludes alternative descriptions. This is what I meant by "model sensitivity". Here, the authors use Generalized Hydrodynamics (GHD) to fit the data. The agreement shows that the GHD framework (which supports Bethe strings) is a viable model for the data. It does not prove that the underlying entities are exclusively Bethe strings, rather than generic short-range bound clusters that would arise in any attractive 1D Bose gas, even in cases where integrability is significantly broken.

3. Stability as a proxy for identity: The authors claim that the stability during 1D expansion "directly probes this hallmark property (long lifetime) of the Bethe strings." While interesting, stability alone is also not a unique identifier. Many-body bound states in 1D are kinematically constrained; a "cluster" of atoms may persist simply due to the lack of available decay channels in a restricted geometry, e.g., solitons, regardless of whether it maps precisely onto a Bethe string solution of the Lieb–Liniger model.

4. Model Dependence: The authors state that they only fit two parameters (initial temperature and crossover temperature). However, the entire interpretation of the resulting energy curve relies on the assumption that the system precisely follows the string-charge distributions of GHD. If the experiment cannot independently verify the string distribution P_n in the manuscript, the claim of "observing" the strings becomes circular: the strings are built into the model to explain the energy, and the energy is then used to claim the observation of the strings. The manuscript undoubtedly presents high-quality experimental work on attractive 1D gases. However, there is a formal scientific distinction between detecting a signature (greater momentum spread) consistent with a theory

and observing the constituent quasi-particles (Bethe strings) of that theory. Regarding the current title, to "Observe Bethe Strings" suggests that one can now directly detect quasi-particles with unique features that are exclusive to Bethe strings. Direct evidence should include either distinct spectral branches or, at minimum, n-body correlations. A good example is the "Experimental Observation of Bethe Strings" in Nature 554 (2018) 219, which achieves this standard.

I reiterate my recommendation: the paper is highly interesting and is suitable for publication if the claim is clarified. Suggested titles include: "Signatures of Bethe string dynamics in an attractive Bose gas far from equilibrium" or "Probing the energy of Bethe strings in a 1D attractive Bose gas."

We thank the referee for his/her continued engagement with our manuscript. In view of the effort the referee has invested, we have revised the title to their second suggestion. Since our work probes not only the energy but also g_2 and other properties of the Bethe strings, we have modified the suggested title slightly to: "Probing Bethe strings in an attractive one-dimensional Bose gas"

Response to reviewer 2&3:

We thank the authors for their iteration of the manuscript, which is now ready for publication.

We thank the referees for the time and effort they have devoted to reviewing our manuscript, and for their recommendation of the work for publication.
